# Genetic insights into elephantgrass persistence for bioenergy purpose

**DOI:** 10.1371/journal.pone.0203818

**Published:** 2018-09-13

**Authors:** João Romero do Amaral Santos de Carvalho Rocha, Tiago de Souza Marçal, Felipe Vicentino Salvador, Adriel Carlos da Silva, Juarez Campolina Machado, Pedro Crescêncio Souza Carneiro

**Affiliations:** 1 Universidade Federal de Viçosa, Campus Universitário, Viçosa, Brazil; 2 Embrapa Gado de Leite, Juiz de Fora, Brazil; Clemson University, UNITED STATES

## Abstract

Persistence may be defined as high sustained yield over multi-harvest. Genetic insights about persistence are essential to ensure the success of breeding programs and any biomass-based project. This paper focuses on assessing the biomass yield persistence for bioenergy purpose of 100 elephantgrass clones measured in six growth seasons in Brazil. To assess the clones' persistence, an index based on random regression models and genotype-ideotype distance was proposed. Results suggested the existence of wide genetic variability between elephantgrass clones, and that the yield trajectories along the harvests generate genetic insights into elephantgrass clones’ persistence and G x E interaction. A gene pool that acts over the biomass yield (regardless of the harvest) was detected, as well as other gene pools, which show differences on genes expression (these genes are the major responsible for clones’ persistence). The lower and higher clones’ persistence was discussed based on genome dosage effect and natural biological nitrogen fixation ability applied to bioenergy industry. The huge potential of energy crops necessarily is associated with genetic insights into persistence, so just this way, breeding programs could breed a new cultivar that fulfills the bioenergy industries.

## Introduction

Elephantgrass [*Pennisetum purpureum* Schumach.; Syn. *Cenchrus purpureus* (Schumach.) Morrone] has potential as a multi-purpose crop, such as the production of bio-based products and co-products and biofuels, besides being used as forage. The nutrient-rich juice can be used as the substrate for fungal-protein production [1] and microbial oil production [2]. The dry biomass can be used to produce chemical composites [3–6], generate energy when burned in boilers [7–9], or convert cellulosic ethanol [10,11].

The use of a given genotype for energy purposes should be mainly based on the knowledge of its calorific value and its yield biomass [12]. Although raw material quality significantly impacts on bioenergy conversion, the greatest economic driver of raw material production is biomass yield. As biomass yield per unit area increases, transport expenses and demand on arable land decreases, leading to an increase in overall economic returns [13].

Besides high biomass yield, biomass energy industries demand a persistent (high sustained yield) and dedicated energy crop cultivar over the harvests. This fact allows better scaling and scheduling of planting and consequently better storage of the raw material, which is given based on the biomass demand, reducing overall costs. Furthermore, due to the perenniality of biomass crop cultivars, they may not be regularly substituted in a plantation [14].

Perennial crops must be sufficiently persistent to maintain their yield performance over the subsequent growth seasons [15–17]. Persistence can be affected by several factors, such as environment (e.g., disease, temperature, drought, etc.) and crop management (e.g., harvest and grazing) [18]. In addition, the genetic contribution for persistence control should be highlighted.

High biomass yield potential and greater output:input ratio are the breeder's goals regarding any dedicated energy crop. Therefore, to achieve the plant ideotype, many steps are involved in the plant breeding process. In any breeding program of energy crops, the phenotyping step will focus on the target traits, i.e., the data are obtained along the yield trajectory in different growth seasons for the same traits, which are denoted by longitudinal data, according to Meyer [19].

Genetics models such as random regression (RR) models deal with longitudinal data very well [20] because they capture the change of a trait continuously over the trajectory with fewer parameters than the multi-trait models [21]. This means that random regression models are a parsimonious covariance structure within a continuous scale (infinite dimensional) that provides estimated genetic values at specific times (harvest) or as a trend over time. Besides that, the RR models partition the variance into genetic and permanent environmental effect without assuming constant during the whole evaluated period [22].

Understanding the yield trajectory along the growth seasons/harvests may determine the success of any biomass-project and even generate genetic insights into elephantgrass clones’ behavior, which is useful for breeding programs. To achieve these highlights, this paper assessed the biomass yield persistence of elephantgrass clones for bioenergy purpose.

## Materials and methods

### Location and experimental conditions

The experiment was carried out at the experimental field of Embrapa Dairy Cattle Research Center, located in the municipality of Coronel Pacheco, MG, Brazil (lat 21° 33’ 18” S, long 43° 15’ 51” W, at 417 m asl), in a red-yellow latosol with the following chemical properties: pH (5.4); H+Al (2.31 cmolc dm^-3^); P and K (1.1 and 23 mg dm^-3^, respectively); and the following exchangeable cations: Al^3+^, Ca^2+^, and Mg^2+^ (0.2, 1.4, and 0.7 cmolc dm^-3^, respectively). The planting was carried out in December 2011, in 0.20 m deep furrows, and 80 kg ha^-1^ P_2_O_5_ fertilizer was applied at planting. After the establishment stage, at 30 days after planting, elephantgrass plots were cut to 0.30 m stubble height (uniformity harvest), beginning the first of the six harvests. Maintenance fertilization was performed with 300 kg ha^-1^ of the N-P_2_O_5_-K_2_O formulation (20:05:20 blended granular fertilizer), after the uniformity harvest and after all harvests. Fertilizers were applied according to the soil analysis.

Six harvests were carried out for this study. Aiming at using them as bioenergetic feedstock, the first (September 28^th^, 2012) and the second (June 04^th^, 2013) harvests were made at 250 growth days; the third harvest (April 15^th^, 2014), at 315 regrowth days. Nevertheless, at the fourth harvest (January 15^th^, 2015), at 275 regrowth days, only the propagation material for the network assay of elephantgrass was collected, i.e., no data field information was registered. At 315 regrowth days, the fifth harvest was performed (November 26^th^, 2015), and the last harvest was carried out at 210 regrowth days (June 22^th^, 2016). Weather and phenotypic data for the term of the present assay are shown in S1 Fig and S1 Table, respectively.

### Experimental design

One hundred genotypes of the Elephantgrass Active Germplasm Bank (BAGCE, S2 Table) were evaluated. Plots (1.5 m x 4 m) consisted of a single 4 m row. Plots were planted side by side, spaced 1.5 m apart and allocated in a 10 x 10 simple lattice design, with two replications.

### Measurement—Biomass yield

The elephantgrass was harvest and weighted in a 3m section from the middle of the rows to obtain the gross fresh biomass weight per plot. Previously, randomly fresh sub-samples of three complete plants from each plot were harvest and weighted (fresh biomass weight) and oven dried at 56°C for 72 hours until reaching constant weight (dry biomass weight). After that, the material was ground until passing through a 1 mm mesh. The dry biomass yield was estimated using the fresh and dry biomass weights of the sub-sample fractions and the fresh biomass weight of the gross sample.

### Statistical analyses

#### Random regression model

Initially, several random regression models were tested to identify the one that best fits the biomass yield trajectory, using the following general model:

$$y_{ijk}=R_{k}+\sum_{m=0}^{M_{b}}\beta_{m}\Phi_{ijm}+\sum_{m=0}^{M}\alpha_{im}\Phi_{ijm}+\sum_{m=0}^{M}p_{ikm}\Phi_{ijm}+e_{ijk}$$

The random regression models were fitted on Legendre polynomials of age at measuring (harvest day) for random and fixed effects, considering various orders of fit. *y*_*ijk*_ is the *i*^th^ genotype measured (*i* = 1, 2, …, *ng*, where *ng* is the total number of genotypes) on the *j*^th^ harvest day (*j* = 1, 2, …, *nh*, where *nh* is the last harvest day) on the *k*^th^ replication. *R*_*k*_ is the fixed effect of replication (*k* = 1, 2). β_*m*_ is the fixed regression coefficient fitted through the quartic degree (order 5 or *M*_*b*_ = 5) of Legendre polynomials to the common average trajectory of all genotypes. The random effects, α_*im*_ and *p*_*ikm*_, are the random regression coefficient for the Legendre polynomial of order *m* for the genetic effect and the permanent environmental effects for the *ik*^th^ plot (*ik* = 1, 2, …, *np*, where *np* is the total number of plots), respectively. Φ_*ijm*_ is the *m*^th^ Legendre polynomial for the *j*^th^ harvest day, standard from -1, to +1, from the *i*^th^ clone; *M* is the fit order, ranging from 1 to 5, of the Legendre polynomial for the genetic and permanent environmental effects, respectively; *e*_*ijk*_ is the residual random effect associated with *y*_*ijk*_. In the matrix notation, the above model is described as follow:

y=Xβ+Zα+Wp+e

where: *y* is the data vector; β is the vector of the effects of the replication (assumed as fixed) added to the overall mean; α is the vector of genetic effects (assumed as random); *p* is the vector of the permanent environment (random); *e* is the vector of residue (random). *X*, *Z*, and *W* represent the incidence matrices for these effects. The fixed part of the model was assumed to account for systematic harvest effect, so that α ~ N(0, *K*_*g*_ ⊗ *I*_*ng*_), α ~ N(0, *K*_*p*_ ⊗ *I*_*np*_), α and *p* are uncorrelated, and *e* ~ N(0, *R*), where *I*_*ng*_ and *I*_*np*_ are identity matrices of appropriated size *ng* and *np*, respectively. ⊗ denotes the direct product. *K*_*g*_ and *K*_*p*_ are the covariance coefficient matrices for genetic and permanent environmental effect, respectively. *R* represents a matrix of residual variances. Several models with different residual variances structures (e.g., unstructured, diagonal, and homogeneous) were tested.

#### Choice of the best-fitted model

The maximum degree of the fitted orthogonal polynomials was tested to determine the most appropriate combination. The random regression models were compared using the likelihood ratio test [23] (LRT) and the Schwarz's Bayesian information criterion (BIC), BIC = -2*LogL* + *pLog*[*n*–*r(x)*], where: *LogL* is the logarithm of the likelihood function; *p* is the number of estimated parameters; *n* is the number of observations, and *r(x)* is the rank of incidence matrix of fixed effect [24].

### Extracting the genetics information

#### Variance components

Based on Kirkpatrick et al. [25], the following estimator was used to obtain the variance components $\left({\hat{V}}_{x}\right)$ on the original scale.

$${\hat{V}}_{x}=\Phi_{ijm}{\hat{K}}_{x}\Phi {'}_{ijm}$$

The term Φ_*ijm*_ was defined in the section Random regression model; ${\hat{K}}_{x}$ is the estimated coefficient covariance matrix for the random effect (genetic or permanent environment).

#### Estimated genetic values

The genetic values $\left({\hat{g}}_{ij}\right)$ were estimated as follows:

$${\hat{g}}_{ij}=\sum_{m=0}^{M}{\hat{\alpha }}_{im}\Phi_{ijm}$$

Where ${\hat{\alpha }}_{im}$ is the random regression coefficient of order *m* for the genetic effects of the *i*^*th*^ clone.

#### Accuracy

Accuracy $\left({\hat{r}}_{ij}\right)$ was estimated as follows:

$${\hat{r}}_{ij}=\sqrt{1-\frac{{\mathrm{PEV}}_{ij}}{{\hat{V}}_{g}}}$$

where PEV_ij_ is the prediction error variance, obtained by the diagonal elements of the transformed coefficient matrix of clone *i* and harvest *j*; ${\hat{V}}_{g}$ is the estimated genetic variance.

#### Eigenfunctions

Additionally, the eigenfunction (Ψ_*i*_) of the genetic coefficient covariance matrix was calculated to provide genetic insights about the studied trait, based on Kirkpatrick et al. [25].

$$\Psi_{i}=\sum_{m=0}^{M}{\left(c_{\Psi i}\right)}_{m}\Phi_{m}$$

where (*c*_Ψi_)_m_ is the *m*^th^ element of the *i*^th^ eigenvector of ${\hat{K}}_{g}$, and Φ_*m*_ is the normalized value of the *m*^th^ Legendre polynomial.

#### Clones’ persistence

Clones' persistence (Persistence_*i*_) was obtained by the distance between each clone in relation to the ideotype (genotype-ideotype distance), considering all estimated genetic values in the range of 250 to 1615 days. The ideotype–max(ĝ_*j*_) was defined as the maximum estimated genetic value in each day in the experimental period. The following algorithm was used:

$${\mathrm{Persistence}}_{i}=\frac{\frac{1}{\sum_{j=250}^{1615}{\left[{\hat{g}}_{ij}-\max\left({\hat{g}}_{j}\right)\right]}^{2}}}{\sum_{i=1}^{100}\left\{\frac{1}{\sum_{j=250}^{1615}{\left[{\hat{g}}_{ij}-\max\left({\hat{g}}_{j}\right)\right]}^{2}}\right\}}\times 100$$

### Software

Statistical analyses were performed using the ASReml 4.1 [26] and R [27] software. The ASReml code is available in S1 Code.

## Results

### The best-fitted model and the general genetic behavior

The goodness of fit of the models is presented in Table 1. According to the Schwarz's Bayesian information criterion (BIC), the best model is denoted by Leg4.1.D with diagonal residual variance and was adopted to describe the changes in the variance and covariance components for elephantgrass biomass yield over multi-harvest. When the models without the genetic or permanent environmental effects were tested by the likelihood ratio test, genetic variability (p-value < 0.01) and significant permanent environmental effect (p-value < 0.01) were detected for all models. All the models run on ASReml follows in S3 Table with the main output.

**Table 1:** Different models fitted with orthogonal Legendre polynomials, number of parameters (p), Schwarz Bayesian information criteria (BIC), and likelihood ratio test (LRT) for genetic and permanent environmental effect.

Model^a^	Fitted order for effect		p	LogL convergence	BIC	LRT (Genetic)	LRT (Perm. env.)
	Genetic	Perm. env.					
Leg3.1.H	3	1	8	Converged	4679.75	188.66**	20.68**
Leg3.2.H	3	2	10	Converged	4693.53	148.88**	20.70**
Leg4.1.H	4	1	12	Converged	4642.74	253.26**	34.26**
Leg4.2.H	4	2	14	Not converged	-	-	-
Leg3.1.D	3	1	12	Converged	4554.36	274.20**	29.88**
Leg3.2.D	3	2	14	Converged	4550.85	221.22**	47.18**
Leg4.1.D	4	1	16	Converged	4549.17	306.98**	32.56**
Leg4.2.D	4	2	18	Converged	4534.36	265.30**	61.16**
Leg3.1.US	3	1	22	Converged	4749.23	185.38**	14.96**
Leg3.2.US	3	2	24	Not converged	-	-	-
Leg4.1.US	4	1	26	Converged	4714.61	247.58**	24.54**
Leg4.2.US	4	2	28	Converged	4728.15	215.34**	24.80**

^a^The models tested are referred to as Leg*m*_*a*_.*m*_*p*._.*x*, where *m*_*a*_ and *m*_*p*_ represent the Legendre's polynomials orders adjusted for genetic and permanent environmental random effects, respectively, and *x* may assume homogeneous (H), diagonal (D) or unstructured (US) residual variance structure.

**significant at 1% by the chi-squared test.

Chi-squared tabulated: 6.63 for 1% significance level.

**Fig 1:**
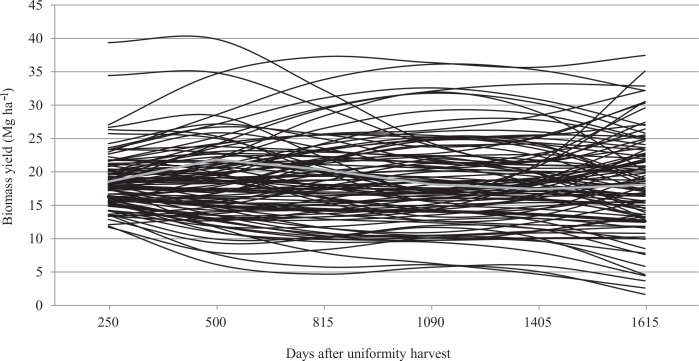
Estimated genetic values for biomass yield over multi-harvest for 100 elephantgrass clones. Each black line represents one clone, and the grey line represents the average biomass yield curve.

Fig 1 shows the general shape of the biomass yield trajectory over the harvests and all random genetic curves. The graph indicates the wide variability that exists around the average curve. Thus, elephantgrass has different biomass yield curves.

### Heritability, genetic variance, phenotypic variance, and permanent variance trajectory for biomass yield over the multi-harvest

**Fig 2:**
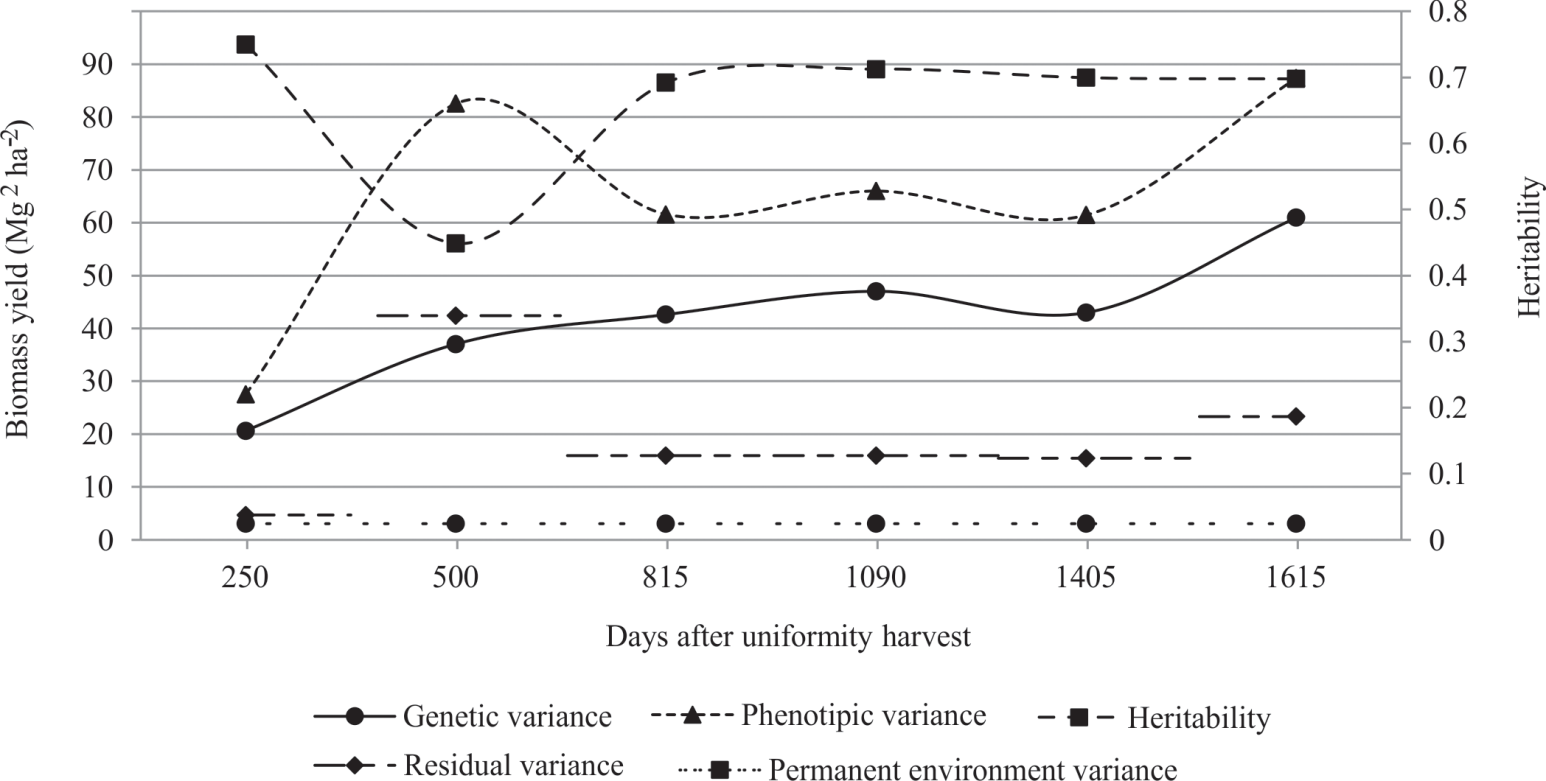
Heritability, genetic and permanent environmental variance, and phenotypic variance trajectory.

Fig 2 shows that the phenotypic variance trajectory was not stable over the multi-harvest. The phenotypic variance reached the peak in the sixth harvest, i.e., the greatest phenotypic variability occurred in the sixth harvest followed by the second one. A constant trajectory was observed for permanent environment variance.

The genetic variance trajectory was increasing over the multi-harvest, with a slight decrease around the fifth harvest (Fig 2). The heritability estimates ranged from 0.45 to 0.75. In general, heritability values decrease from the first to the second harvest (indicating that the second harvest is strongly influenced by the environment) and remain above 0.69 from the third harvest onwards (Fig 2). In addition, the genetic values for the fourth harvest (1090 days, without phenotypic data) were predicted with 84.41% average accuracy.

All trajectories were estimated from the random regression model (Leg4.1.D) fitted by Legendre polynomials for biomass yield over multi-harvest. See S2 Fig for accumulated rainfall and temperature data.

**Fig 3:**
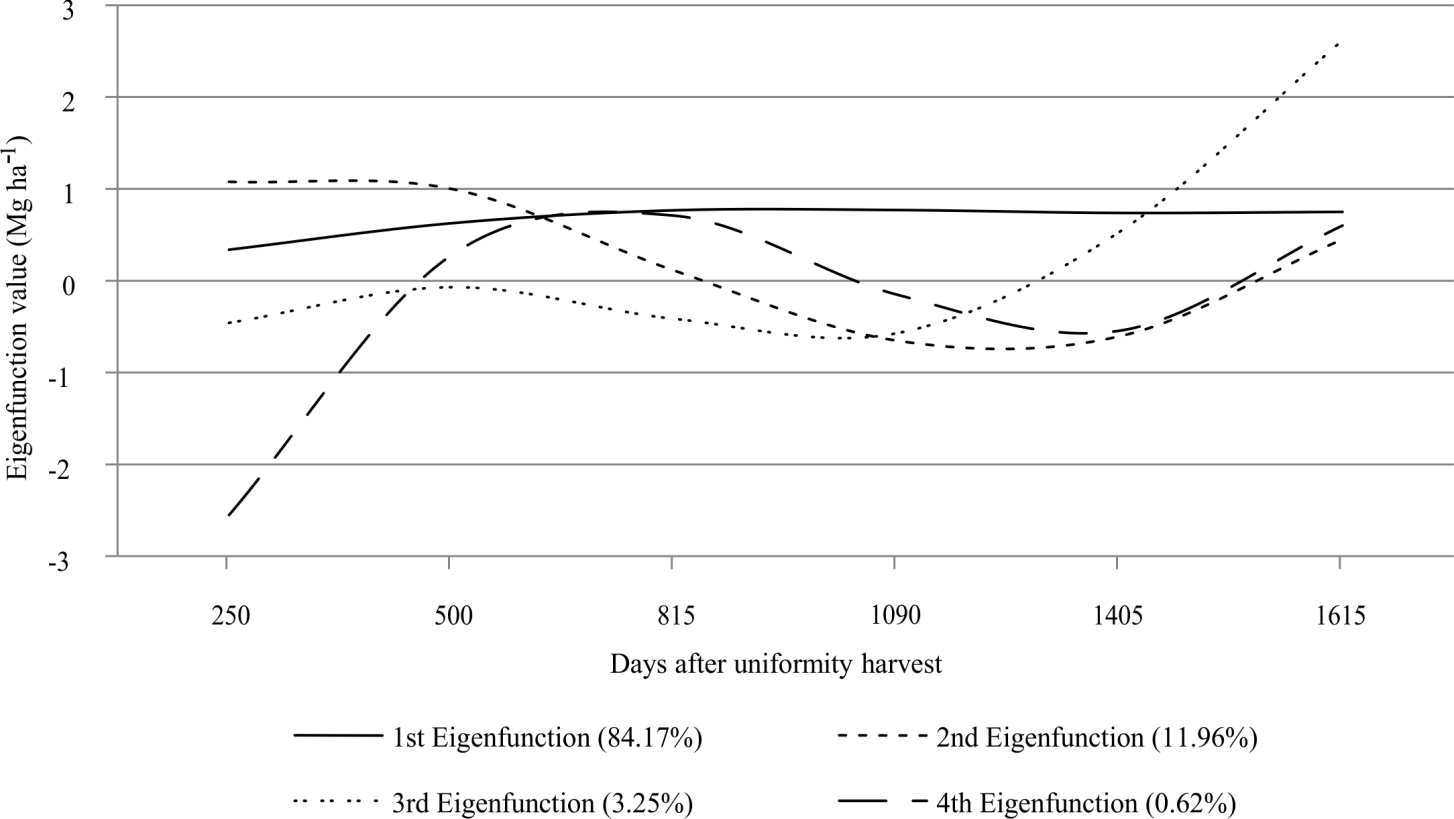
Estimates of the four eigenfunctions. Their proportional eigenvalues for the genetic covariance function are in parentheses.

Fig 3 reveals that the first eigenfunction (associated with the largest eigenvalue) is nearly constant over the multi-harvest, indicating that 86% of the genetic variation is explained by a gene pool that acts over the biomass yield, regardless of the growth season.

The second eigenfunction (around 11% of the genetic variation, Fig 3) represents other gene pool that shows differences in genes expression under different environment conditions, i.e., biomass yield reverses between the first two harvests (250 and 500 days) in relation to the third, fourth, and fifth harvests (815, 1090, and 1405 days) and reverses again in relation to the last one (1615 days). This genetic factor is the major responsible for the genotypes by environments (growth seasons) interaction. The third and fourth eigenfunction was not interpreted due to their small genetic variation proportion (3.31 and 0.26%, Fig 3).

### Elephantgrass clones’ persistence

**Fig 4:**
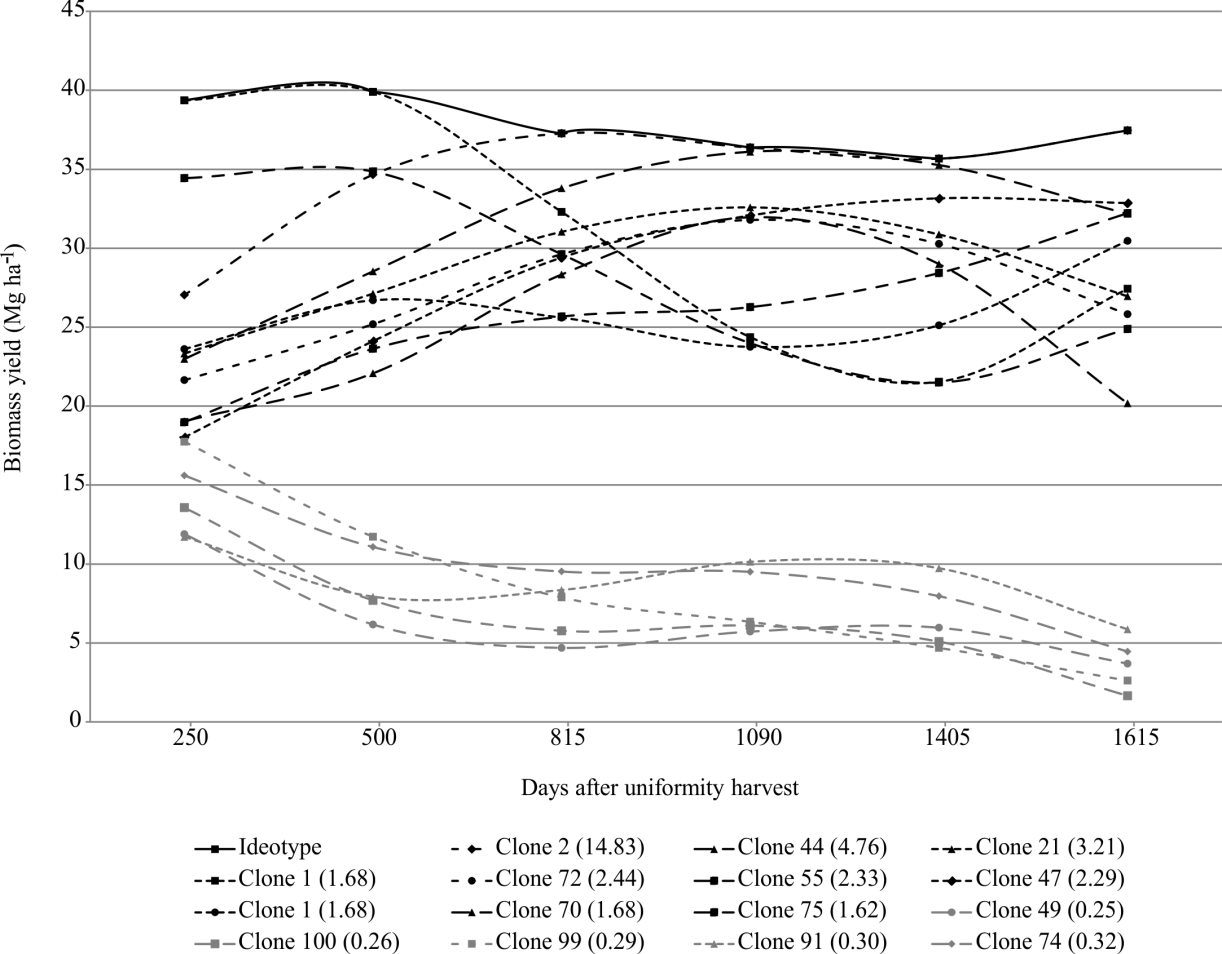
Elephantgrass biomass yield trajectory over the multi-harvest. The ten most persistent and the five least persistent clones. Persistence values are in parentheses.

The experimental biomass yield means were 12.51, 29.60, 19.60, 15.28, and 19.07. Mg ha^-1^ for 250, 500, 815, 1405 and 1615 days after uniformity harvest, respectively. Fig 4 shows the ten most and the five least persistent elephantgrass clones. Clone 2 was not the most yielded at all harvests; however, it sustained the biomass yield with the highest performance from the third harvest (815 days) onwards.

Among the ten most persistent clones, clones 95 and 55 showed the highest biomass yield in the first two harvests, i.e., these clones achieved the biomass yield peak quickly, but were not able to persist over the multi-harvest (Fig 4). Clones 49, 100 and 99 showed the lowest persistence (Fig 4). Clones 99 and 100 were the only ones that completely died in the plots of the first replication in the fifth harvest (1405 days) and in all plots in the sixth harvest (1615 days).

## Discussions

### The best-fitted model

Statistical methods for analyzing data of perennials need to appropriately model the genetic effects over time [28]. A suitable method for modeling the genetic trajectory over time is the random regression model, as commonly used in the animal sciences [20, 22]. It is noteworthy that the experimental data used in this work shows unequally spaced sample intervals over the harvests (250, 500, 815, 1090, 1405, 1615 days after the uniformity harvest) and that no phenotypic data was collected in the fourth harvest (at 1090 days after the uniformity harvest). Kirkpatrick et al. [25] relate that, under this condition, the random regression models are sufficient, being the adequate methodology.

Different criteria have been used to find the polynomial order of the model with the best fit and parsimony [29]. In the present study, the best-fitted model was indicated by the BIC criterion (Legendre polynomials of the fourth order, for genetic effect, and first order, for permanent environmental effect with diagonal residual variance structure–Leg4.1.D, Table 1). From this model, all interpretations were performed.

Simple repeatability and multi-trait models can be employed for longitudinal data analysis [21]. However, the repeatability model considers the genetic correlation between the different harvests equal to one, i.e., exactly the same genes acting in the control of the trait over time [30]. The repeatability model can be reproduced from the random regression model, considering the fitted order equal to one (intercept random regression model). Thus, the repeatability model is not suitable to represent the genetic behavior presented by the biomass yield over the multi-harvest. The multi-trait model assumes that the data are discontinuous, while in the present work the harvests are taken continuously. Therefore, the extrapolation of the genetic value to an unobserved harvest is not recommended. The multi-trait model would need to estimate 30 parameters, while the chosen random regression model (Leg4.1.D) required only 16 parameters, thus being parsimonious, in addition, the multi-trait model does not allow computing the random permanent environment effects (i.e., the permanent environment effect is confused or overlaid with the temporary environment effect).

### Genetic variability and general genetic behavior

The high amplitude that the random genetic curves deviations showed in relation to general biomass yield trajectory suggests high genetic variability (Fig 1). Wide genetic variability in the studied clones was expected since they are clones belonging to a germoplasm bank (BAGCE) and have not yet been genetically improved for bioenergetics [8]. According to Azevedo et al. [31] and Rocha et al. [9], these clones presented genetic variability using single sequence repeated markers and the biomass yield trait, respectively. Knowledge about genetic diversity is the key to further improvement, and evaluation of diversity in germplasm is essential for the effective use of genetic resources in breeding programs. Assessing the diversity information would facilitate the progress in plant breeding [32].

### Permanent environment, plasticity, and persistence

Besides the genetic and the temporary environmental effect that composes a phenotype, a permanent environmental effect occurs in longitudinal data. Permanent implies stability and a constant or common presence to repeated measures [33, 34]. Kruuk and Hadfield [33] have shown that permanent environmental effect may overlap with several factors, e.g., dominance or epistatic genetic effects, maternal genetic effects, common environmental effects, especially the phenotypic plasticity. It is worth mentioning that the permanent environmental effect is estimated by the variance among repeated measures in the same individual (i.e., in animal science no replications of the individual is used in the experiment as in plant science). Replications of individuals or families are very common in plant breeding experiments, and the permanent environmental effect is estimated by the variance among repeated measures in the plots (different genotypes-replications combination). Under this condition, additional effects occur due to differential competition between the same individual in different plots (due to experimental randomization).

The second harvest showed the greatest phenotypic variance and the greatest temporary environment or residual variance (Fig 2); this behavior may be explained by the rainfall and temperature data (S2 Fig). The second harvest showed the most favorable environmental condition (e.g., temperature and rainfall, see S2 Fig) for elephantgrass growth, which is confirmed by the highest biomass mean, 29.60 Mg ha^-1^ (51% more productive than the second most productive harvest). Thus, resources availability (e.g., light, water, nutrient, temperature, etc.) can stimulate phenotypic plastic response subject to generate a large phenotypic variance.

According to Nicotra et al. [35], phenotypic plasticity is the range of phenotypes that a single genotype can express as a function of its environment. Phenotypic plasticity depends on the genome plasticity, defined as a change in the genome structure or organization associated with environmental signals [35]. In this context, the phenotypic plasticity can be considered as favorable or unfavorable changes for genotype adaptedness [36]. Nicotra et al. [35] define adaptive plasticity as the phenotypic plasticity that increases the global fitness of a genotype. In the plant breeding context, the adaptive plasticity is equivalent to the adaptability proposed by Finlay and Wilkinson [37].

Bradshaw [38] relates that plasticity is related to stability. Plasticity can be a simple sign of weakness (of lack of fitness), but it can also be a sign of strength, attributed to maintenance mechanisms of fitness. Breeders attempt to select genotypes with consistent performance between a range of target environments in order to reduce the G x E effects [39]. For instance, breeders try to produce cultivars that reliably perform despite year-to-year fluctuations in weather patterns. In the case where limited phenotypic plastic response confers stability, the low G × E contribution may have a desirable effect by enabling germplasm to predictably perform across environments [39]. Plasticity not only gives an edge over competitors but also is essential for genotype persistence in new or changing environments [40]. In the plant breeding scenario, persistence was defined as high sustained yield over environmental changes [15–17] and is a relevant trait under several aspects of the bioenergy industry.

### Insights about G x E interaction—Driving the selection

When trajectory curves are non-constant, genotypes show plasticity (in new and changing environments–growth seasons), and when the curves intersect, a G x E interaction occurs (see Fig 4). According to van Eeuwijk et al. [36], this type of G x E has more severe consequences for breeders as it will change the rank order of clones in function of the environmental conditions.

The main reason for the seasonality of elephantgrass yield over multi-harvest is the differential genes expression, i.e., the environmental effect promotes different levels of genes expression (even the nonexpression of the genes) that affect the elephantgrass biomass yield. The differential genes expression is the theoretical base of G x E interaction. The first eigenfunction (Fig 3) captured a gene pool that was equally expressed in all growth seasons (e.g., general adaptability genes). The second eigenfunction (Fig 3) clustered genes that expressed themselves depending on the environmental differences (these genes determine the persistence—specific adaptability genes). The uninterpreted eigenfunctions showed small eigenvalues (Fig 3), and according to Kirkpatrick et al. [25], eigenfunctions with very small (or null) eigenvalues represent deformations for which there is little (or none) genetic variation.

The second harvest is not recommended for selection (h^2^ = 0.45, Fig 2). Results also indicated that the first, third, fourth, fifth, and sixth harvests represent a more favorable scenario for selection (i.e., accuracy higher than 83%). The genetic breeding must handle inheritable traits, i.e., those with high heritability. The heritability of a trait will have an impact on selection decisions. Genetic progress tends to be much slower in lowly heritable traits. Conversely, with higher heritability, a faster progress is achieved with selection due to greater accuracy in selection decisions [41].

Forage breeding can be a complex task due to the plant perenniality, among several other factors [42]. Persistence is a complex trait affected by a large number of biotic, abiotic, and genetic factors, e.g., diseases and pests, mechanical harvesting equipment, intensity of harvest management, temperature, drought, plant competition [18], and genome plasticity. Thus, pyramiding of genes that express themselves in different environments would increase genome plasticity and consequently increase the genotypes’ persistence.

### Lowest persistence—Supported by the genome dosage

The death of clones 99 and 100 in the last two harvests is a factor that explains the lowest persistence. Death may have been caused by the low perenniality of these clones, i.e., the genome dosage may interfere with the perenniality of elephantgrass clones.

Elephantgrass is allotetraploid (2n = 4x = 28, A'A'BB) with ploidy level variations [43]. Pearl millet [*Pennisetum Glaucum* (L.) R. Br.; Syn. *Cenchrus americanus* (L.) Morrone, 2n = 2x = 14, AA] has an annual growth habit and can produce interspecific hybrids with elephantgrass [43, 44]. For instance, clone 99 is triploid (2n = 3x = 21, AA'B), and clone 100 is hexaploid (2n = 6x = 42, AAA'A'BB). Triploids have an additional copy of A pearl millet genome, whereas the hexaploids have two copies of A pearl millet genome. The A' genome chromosomes are larger than the B genome chromosomes. Moreover, the B genome contributes to elephantgrass perennial life cycle [45]. However, the additional genome dose of the pearl millet (A genome in Clones 99 and 100) may reduce the perenniality due to the annual growth habit genes present in the A genome.

Clone 46 (Kizozi) also showed low persistence. Kizozi was previously studied by Techio et al. [46] and confirmed as a wild species of the genus *Cenchrus* due to the somatic chromosome number (2n = 54). Wild species could have less adaptive genes when compared with breeding cultivars, which leads them to low persistence. Some of the least persistent clones are tetraploid (e.g., clones 30, 34, 35, 6, 77, 74, 91, and 49). The low persistence presented by clone 49 (Mott) is due to its reduced plant height (dwarfing genes). Mott is specifically adapted to be used as forage, in the pasture, owing to its high nutritive value, and it has previously been identified as of low potential for bioenergy production [9]. The other clones showed reduced biomass yield, low height, thin stalks [8], and consequently low persistence. These clones were classified as Napier, Mercker, or intermediate (Napier/Mercker) group [47], and were studied by Rocha et al. [9] regarding the aptitude for bioenergy purposes. The authors showed that Napier, Mercker, and intermediate groups have a low potential for biomass energy production.

Furthermore, biomass crops should be perennial because a cultivar cannot be regularly substituted in a plantation. Perenniality, unlike annual habit, would be advantageous due to costs reduction with the establishment of energy crops [14].

### Clones’ persistence applied to bioenergy industries

The aim of supplying biomass to bioenergy is to achieve high energy yields per unit area and the best possible fuel quality. The energy yield comprises the biomass yield and the energy content of the biomass. Fuel quality is determined by the physical and chemical properties and influences the entire process of thermal utilization [48]. However, the greatest economic driver of raw material production is biomass yield [13]. Besides high energy yields per area, biomass energy industries look for a cultivar with higher persistence (high sustained yield). This fact allows reducing costs due to the better scaling and scheduling of planting, harvesting, and storing of the raw material, based on the biomass demand.

Persistence is an economically important trait for perennial forages due to the costs involved in sward establishment. This trait is dependent on the vigor of a plant and its ability to survive and contribute to yield and ground cover [16], and thus, the clumps expansion capacity and the number of basal and axillary tillers may directly impact on persistence. The persistence of elephantgrass clones was measured using clones-ideotype distance over multi-harvest. This approach takes into account the yield stability and the high genetic values.

Clone 2 showed the highest sustained biomass yield over multi-harvest (highest persistence), which may be supported by the natural biological nitrogen fixation (BNF) ability of this clone, as reported by Morais et al. [49]. The higher natural nitrogen input (e.g., BFN) is related to higher biomass production and competitive advantages, especially under unfavorable environmental conditions. Furthermore, to make the input:output rate more favorable to energy balance, industrial nitrogen inputs (N fertilizers) must be minimized. Low nitrogen requirement is desirable not only for being a valued constituent in terms of conversion to energy but also for N fertilizer is a costly input [50].

Understanding the yield trajectories patterns of elephantgrass clones allowed detecting the G x E interaction and assess the persistence of these plants. The eigenfunctions indicate valuable insights about the G x E interaction, i.e., there is a gene pool (general adaptability genes) that is expressed in all growth seasons and another gene pool (specific adaptability genes or persistence genes) that is expressed under different environmental conditions. These findings suggest that increase the elephantgrass persistence can be successfully achieved with breeding efforts, as a consequence of wide genetic variability for biomass yield and high heritability values over harvests. Moreover, the random regression model allows optimizing elephantgrass management techniques, as well as developing strategies for crosses (i.e., explore the genetic variability).

Future persistence studies applied to elephantgrass should integrate molecular markers information, besides the phenotypic data, aiming at finding several stable quantitative trait loci (QTLs) across multi-harvest. However, the instable QTLs detected may contribute to the persistence increment by recombining (genes pyramiding) unstable QTLs. QTLs studies would allow identifying the genetic basis of G x E in the form of QTLs x E interaction [36]. In addition, further studies on natural biological fixation nitrogen vs. persistence specifically designed for bioenergy purposes must be developed, mainly to keep a favorable energy balance and reduce costs with N fertilizer input. In this way, breeders will be able to rationally deal with the factors that determine persistence by using these factors in their favor. Moreover, they will breed cultivars that will be adopted in bioenergy industries, mainly due to the increase in persistence.

## Supporting information

S1 Fig Rainfall (bars) and average temperature (lines) during the current assay and historical data for 30 years. (TIF)

S2 Fig Accumulated rainfall (black bars) and average temperature (grey bars) with the maximum and minimum average temperature (deviation lines) during the multi-harvest. (TIF)

S1 Table Elephantgrass phenotypic data (biomass yield) collected in field experiment from five harvests. (DOCX)

S2 Table Registrations names of clones of the Active Elephantgrass Germplasm Bank (BAGCE) maintained by Embrapa Dairy Cattle Research Center and their respective code. (DOCX)

S3 Table ASReml output of all models tested. (DOCX)

S1 Code ASReml code. (DOCX)
