# Supplementary figures and images for: Genetic insights into elephantgrass persistence for bioenergy purpose
Source: PLoS One. 2018 Sep 13;13(9):e0203818. doi: 10.1371/journal.pone.0203818 (PMC6136769; doi:10.1371/journal.pone.0203818)

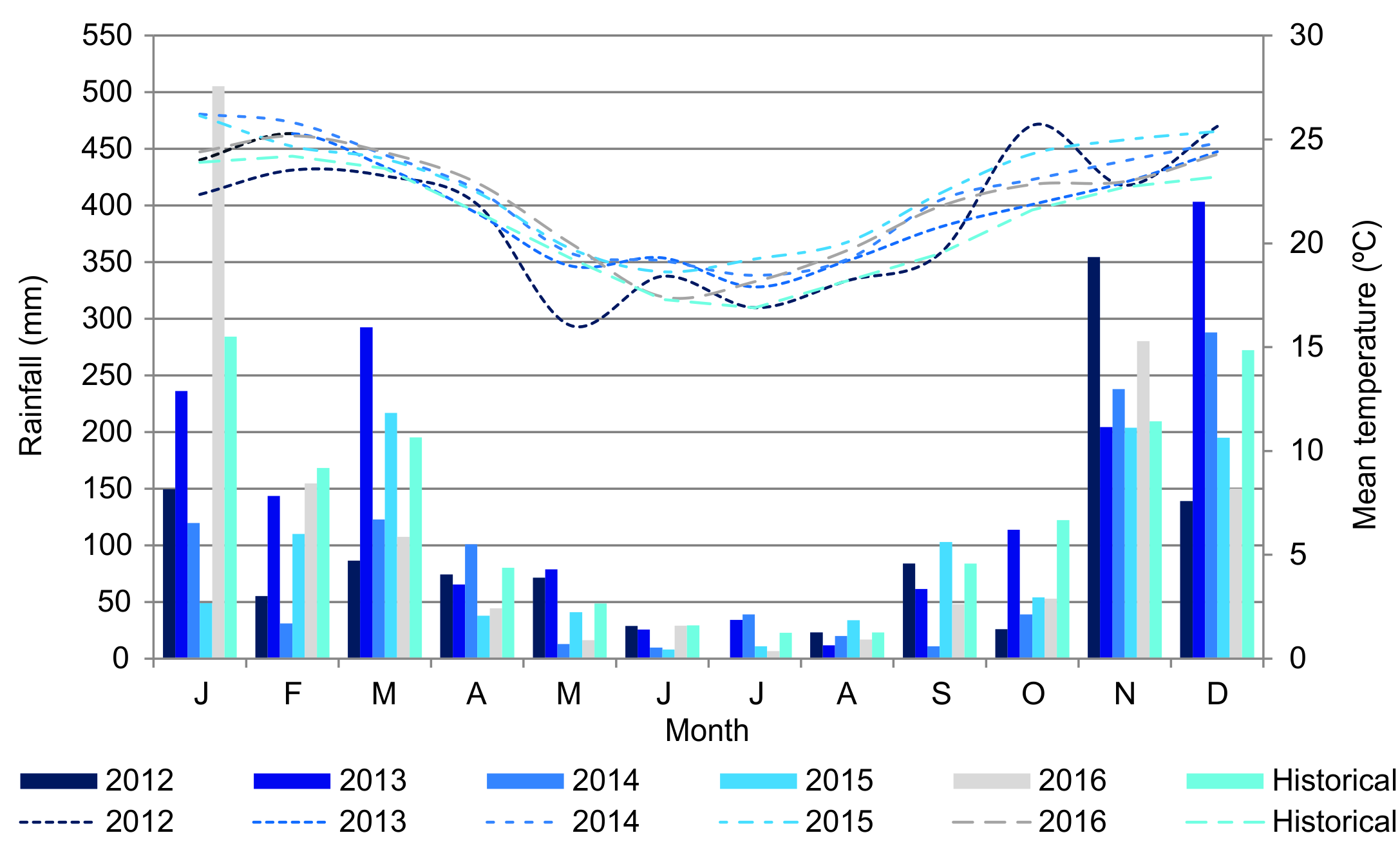

Supplement: S1 Fig — (TIF) [file pone.0203818.s001.tif]

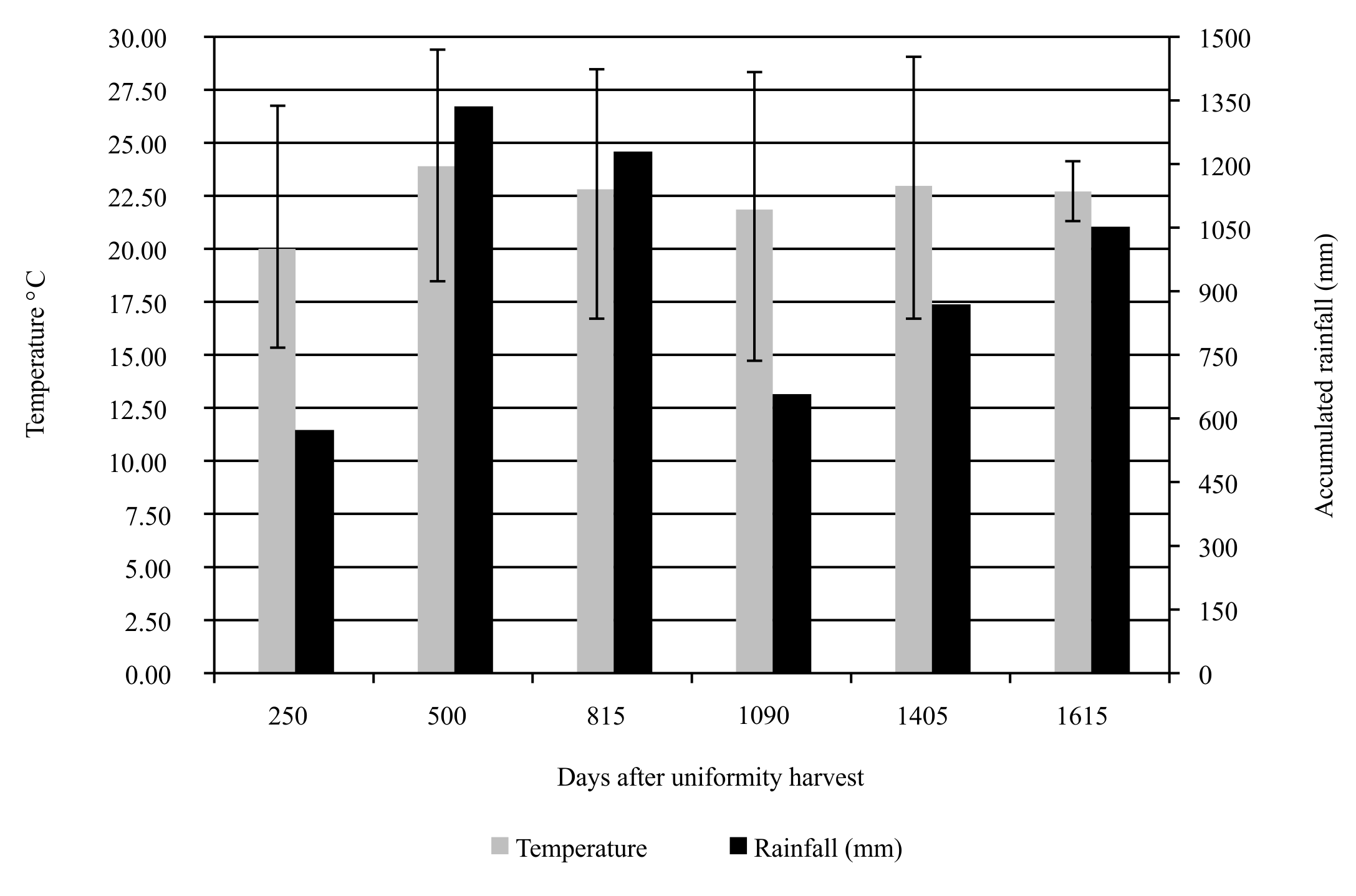

Supplement: S2 Fig — (TIF) [file pone.0203818.s002.tif]
