# Supplementary material for: Genetic insights into elephantgrass persistence for bioenergy purpose
Source: PLoS One. 2018 Sep 13;13(9):e0203818. doi: 10.1371/journal.pone.0203818 (PMC6136769; doi:10.1371/journal.pone.0203818)
Supplement: S1 Table — (DOCX) [file pone.0203818.s003.docx]

**S1 Table.** Elephantgrass phenotypic data (biomass yield) collected on field experiment from five harvests.

| Harv | Id | Gen | HarvRep | Plot | Int | Block | Rep | BY | Day |
| --- | --- | --- | --- | --- | --- | --- | --- | --- | --- |
| 1 | 1 | 1 | 1 | 1 | 11 | 1 | 1 | 19.68 | 250 |
| 1 | 2 | 2 | 1 | 2 | 12 | 1 | 1 | 20.04 | 250 |
| 1 | 3 | 3 | 1 | 3 | 13 | 1 | 1 | 12.92 | 250 |
| 1 | 4 | 4 | 1 | 4 | 14 | 1 | 1 | 13.15 | 250 |
| 1 | 5 | 5 | 1 | 5 | 15 | 1 | 1 | 13.36 | 250 |
| 1 | 6 | 6 | 1 | 6 | 16 | 1 | 1 | 6.98 | 250 |
| 1 | 7 | 7 | 1 | 7 | 17 | 1 | 1 | 17.02 | 250 |
| 1 | 8 | 8 | 1 | 8 | 18 | 1 | 1 | 8.99 | 250 |
| 1 | 9 | 9 | 1 | 9 | 19 | 1 | 1 | 13.03 | 250 |
| 1 | 10 | 10 | 1 | 10 | 110 | 1 | 1 | 14.58 | 250 |
| 1 | 11 | 11 | 1 | 11 | 111 | 2 | 1 | 16.63 | 250 |
| 1 | 12 | 12 | 1 | 12 | 112 | 2 | 1 | 13.8 | 250 |
| 1 | 13 | 13 | 1 | 13 | 113 | 2 | 1 | 10.67 | 250 |
| 1 | 14 | 14 | 1 | 14 | 114 | 2 | 1 | 10.21 | 250 |
| 1 | 15 | 15 | 1 | 15 | 115 | 2 | 1 | 13.3 | 250 |
| 1 | 16 | 16 | 1 | 16 | 116 | 2 | 1 | 8.73 | 250 |
| 1 | 17 | 17 | 1 | 17 | 117 | 2 | 1 | 10.67 | 250 |
| 1 | 18 | 18 | 1 | 18 | 118 | 2 | 1 | 8.01 | 250 |
| 1 | 19 | 19 | 1 | 19 | 119 | 2 | 1 | 19.49 | 250 |
| 1 | 20 | 20 | 1 | 20 | 120 | 2 | 1 | 13.85 | 250 |
| 1 | 21 | 21 | 1 | 21 | 121 | 3 | 1 | 19.36 | 250 |
| 1 | 22 | 22 | 1 | 22 | 122 | 3 | 1 | 10.81 | 250 |
| 1 | 23 | 23 | 1 | 23 | 123 | 3 | 1 | 10.71 | 250 |
| 1 | 24 | 24 | 1 | 24 | 124 | 3 | 1 | 13.24 | 250 |
| 1 | 25 | 25 | 1 | 25 | 125 | 3 | 1 | 8.99 | 250 |
| 1 | 26 | 26 | 1 | 26 | 126 | 3 | 1 | 13.07 | 250 |
| 1 | 27 | 27 | 1 | 27 | 127 | 3 | 1 | 11.64 | 250 |
| 1 | 28 | 28 | 1 | 28 | 128 | 3 | 1 | 13.09 | 250 |
| 1 | 29 | 29 | 1 | 29 | 129 | 3 | 1 | 9.39 | 250 |
| 1 | 30 | 30 | 1 | 30 | 130 | 3 | 1 | 10.32 | 250 |
| 1 | 31 | 31 | 1 | 31 | 131 | 4 | 1 | 19.76 | 250 |
| 1 | 32 | 32 | 1 | 32 | 132 | 4 | 1 | 13.27 | 250 |
| 1 | 33 | 33 | 1 | 33 | 133 | 4 | 1 | 12.49 | 250 |
| 1 | 34 | 34 | 1 | 34 | 134 | 4 | 1 | 9.48 | 250 |
| 1 | 35 | 35 | 1 | 35 | 135 | 4 | 1 | 9.12 | 250 |
| 1 | 36 | 36 | 1 | 36 | 136 | 4 | 1 | 16.25 | 250 |
| 1 | 37 | 37 | 1 | 37 | 137 | 4 | 1 | 22.92 | 250 |
| 1 | 38 | 38 | 1 | 38 | 138 | 4 | 1 | 12.55 | 250 |
| 1 | 39 | 39 | 1 | 39 | 139 | 4 | 1 | 22.46 | 250 |
| 1 | 40 | 40 | 1 | 40 | 140 | 4 | 1 | 14.19 | 250 |
| 1 | 41 | 41 | 1 | 41 | 141 | 5 | 1 | 11.85 | 250 |
| 1 | 42 | 42 | 1 | 42 | 142 | 5 | 1 | 23.01 | 250 |
| 1 | 43 | 43 | 1 | 43 | 143 | 5 | 1 | 12.78 | 250 |
| 1 | 44 | 44 | 1 | 44 | 144 | 5 | 1 | 19.71 | 250 |
| 1 | 45 | 45 | 1 | 45 | 145 | 5 | 1 | 9.67 | 250 |
| 1 | 46 | 46 | 1 | 46 | 146 | 5 | 1 | 11.26 | 250 |
| 1 | 47 | 47 | 1 | 47 | 147 | 5 | 1 | 12.12 | 250 |
| 1 | 48 | 48 | 1 | 48 | 148 | 5 | 1 | 13 | 250 |
| 1 | 49 | 49 | 1 | 49 | 149 | 5 | 1 | 5.06 | 250 |
| 1 | 50 | 50 | 1 | 50 | 150 | 5 | 1 | 11.24 | 250 |
| 1 | 51 | 51 | 1 | 51 | 151 | 6 | 1 | 17.75 | 250 |
| 1 | 52 | 52 | 1 | 52 | 152 | 6 | 1 | 9.66 | 250 |
| 1 | 53 | 53 | 1 | 53 | 153 | 6 | 1 | 16.23 | 250 |
| 1 | 54 | 54 | 1 | 54 | 154 | 6 | 1 | 14.54 | 250 |
| 1 | 55 | 55 | 1 | 55 | 155 | 6 | 1 | 34.46 | 250 |
| 1 | 56 | 56 | 1 | 56 | 156 | 6 | 1 | 9.42 | 250 |
| 1 | 57 | 57 | 1 | 57 | 157 | 6 | 1 | 17.86 | 250 |
| 1 | 58 | 58 | 1 | 58 | 158 | 6 | 1 | 13.06 | 250 |
| 1 | 59 | 59 | 1 | 59 | 159 | 6 | 1 | 15.51 | 250 |
| 1 | 60 | 60 | 1 | 60 | 160 | 6 | 1 | 16.73 | 250 |
| 1 | 61 | 61 | 1 | 61 | 161 | 7 | 1 | 10.38 | 250 |
| 1 | 62 | 62 | 1 | 62 | 162 | 7 | 1 | 12.95 | 250 |
| 1 | 63 | 63 | 1 | 63 | 163 | 7 | 1 | 9.62 | 250 |
| 1 | 64 | 64 | 1 | 64 | 164 | 7 | 1 | 9.55 | 250 |
| 1 | 65 | 65 | 1 | 65 | 165 | 7 | 1 | 8.61 | 250 |
| 1 | 66 | 66 | 1 | 66 | 166 | 7 | 1 | 8.87 | 250 |
| 1 | 67 | 67 | 1 | 67 | 167 | 7 | 1 | 12.7 | 250 |
| 1 | 68 | 68 | 1 | 68 | 168 | 7 | 1 | 4.86 | 250 |
| 1 | 69 | 69 | 1 | 69 | 169 | 7 | 1 | 9.63 | 250 |
| 1 | 70 | 70 | 1 | 70 | 170 | 7 | 1 | 9.99 | 250 |
| 1 | 71 | 71 | 1 | 71 | 171 | 8 | 1 | 10.12 | 250 |
| 1 | 72 | 72 | 1 | 72 | 172 | 8 | 1 | 17.59 | 250 |
| 1 | 73 | 73 | 1 | 73 | 173 | 8 | 1 | 10.25 | 250 |
| 1 | 74 | 74 | 1 | 74 | 174 | 8 | 1 | 7.64 | 250 |
| 1 | 75 | 75 | 1 | 75 | 175 | 8 | 1 | 12.52 | 250 |
| 1 | 76 | 76 | 1 | 76 | 176 | 8 | 1 | 10.46 | 250 |
| 1 | 77 | 77 | 1 | 77 | 177 | 8 | 1 | 4.86 | 250 |
| 1 | 78 | 78 | 1 | 78 | 178 | 8 | 1 | 10.91 | 250 |
| 1 | 79 | 79 | 1 | 79 | 179 | 8 | 1 | 15.56 | 250 |
| 1 | 80 | 80 | 1 | 80 | 180 | 8 | 1 | 14.45 | 250 |
| 1 | 81 | 81 | 1 | 81 | 181 | 9 | 1 | 8.39 | 250 |
| 1 | 82 | 82 | 1 | 82 | 182 | 9 | 1 | 14.35 | 250 |
| 1 | 83 | 83 | 1 | 83 | 183 | 9 | 1 | 4.99 | 250 |
| 1 | 84 | 84 | 1 | 84 | 184 | 9 | 1 | 16.23 | 250 |
| 1 | 85 | 85 | 1 | 85 | 185 | 9 | 1 | 10.94 | 250 |
| 1 | 86 | 86 | 1 | 86 | 186 | 9 | 1 | 12 | 250 |
| 1 | 87 | 87 | 1 | 87 | 187 | 9 | 1 | 6.55 | 250 |
| 1 | 88 | 88 | 1 | 88 | 188 | 9 | 1 | 12.89 | 250 |
| 1 | 89 | 89 | 1 | 89 | 189 | 9 | 1 | 8.43 | 250 |
| 1 | 90 | 90 | 1 | 90 | 190 | 9 | 1 | 5.21 | 250 |
| 1 | 91 | 91 | 1 | 91 | 191 | 10 | 1 | 6.13 | 250 |
| 1 | 92 | 92 | 1 | 92 | 192 | 10 | 1 | 7.19 | 250 |
| 1 | 93 | 93 | 1 | 93 | 193 | 10 | 1 | 16.56 | 250 |
| 1 | 94 | 94 | 1 | 94 | 194 | 10 | 1 | 9.04 | 250 |
| 1 | 95 | 95 | 1 | 95 | 195 | 10 | 1 | 39.26 | 250 |
| 1 | 96 | 96 | 1 | 96 | 196 | 10 | 1 | 21.17 | 250 |
| 1 | 97 | 97 | 1 | 97 | 197 | 10 | 1 | 12.15 | 250 |
| 1 | 98 | 98 | 1 | 98 | 198 | 10 | 1 | 14.18 | 250 |
| 1 | 99 | 99 | 1 | 99 | 199 | 10 | 1 | 10.21 | 250 |
| 1 | 100 | 100 | 1 | 100 | 1100 | 10 | 1 | 5.26 | 250 |
| 1 | 101 | 1 | 6 | 101 | 11 | 11 | 2 | 17.18 | 250 |
| 1 | 102 | 2 | 6 | 102 | 12 | 12 | 2 | 21.87 | 250 |
| 1 | 103 | 3 | 6 | 103 | 13 | 13 | 2 | 13.49 | 250 |
| 1 | 104 | 4 | 6 | 104 | 14 | 14 | 2 | 7.32 | 250 |
| 1 | 105 | 5 | 6 | 105 | 15 | 15 | 2 | 12.33 | 250 |
| 1 | 106 | 6 | 6 | 106 | 16 | 16 | 2 | 7.99 | 250 |
| 1 | 107 | 7 | 6 | 107 | 17 | 17 | 2 | 11.29 | 250 |
| 1 | 108 | 8 | 6 | 108 | 18 | 18 | 2 | 7.33 | 250 |
| 1 | 109 | 9 | 6 | 109 | 19 | 19 | 2 | 11.37 | 250 |
| 1 | 110 | 10 | 6 | 110 | 110 | 20 | 2 | 9.69 | 250 |
| 1 | 111 | 11 | 6 | 111 | 111 | 11 | 2 | 9.32 | 250 |
| 1 | 112 | 12 | 6 | 112 | 112 | 12 | 2 | 10.86 | 250 |
| 1 | 113 | 13 | 6 | 113 | 113 | 13 | 2 | 8.12 | 250 |
| 1 | 114 | 14 | 6 | 114 | 114 | 14 | 2 | 9.89 | 250 |
| 1 | 115 | 15 | 6 | 115 | 115 | 15 | 2 | 11.96 | 250 |
| 1 | 116 | 16 | 6 | 116 | 116 | 16 | 2 | 4.65 | 250 |
| 1 | 117 | 17 | 6 | 117 | 117 | 17 | 2 | 6.5 | 250 |
| 1 | 118 | 18 | 6 | 118 | 118 | 18 | 2 | 13.4 | 250 |
| 1 | 119 | 19 | 6 | 119 | 119 | 19 | 2 | 14.57 | 250 |
| 1 | 120 | 20 | 6 | 120 | 120 | 20 | 2 | 9.4 | 250 |
| 1 | 121 | 21 | 6 | 121 | 121 | 11 | 2 | 17.04 | 250 |
| 1 | 122 | 22 | 6 | 122 | 122 | 12 | 2 | 6.78 | 250 |
| 1 | 123 | 23 | 6 | 123 | 123 | 13 | 2 | 8.26 | 250 |
| 1 | 124 | 24 | 6 | 124 | 124 | 14 | 2 | 9.91 | 250 |
| 1 | 125 | 25 | 6 | 125 | 125 | 15 | 2 | 11.67 | 250 |
| 1 | 126 | 26 | 6 | 126 | 126 | 16 | 2 | 12.15 | 250 |
| 1 | 127 | 27 | 6 | 127 | 127 | 17 | 2 | 11.64 | 250 |
| 1 | 128 | 28 | 6 | 128 | 128 | 18 | 2 | 11.5 | 250 |
| 1 | 129 | 29 | 6 | 129 | 129 | 19 | 2 | 8.37 | 250 |
| 1 | 130 | 30 | 6 | 130 | 130 | 20 | 2 | 7.97 | 250 |
| 1 | 131 | 31 | 6 | 131 | 131 | 11 | 2 | 15.75 | 250 |
| 1 | 132 | 32 | 6 | 132 | 132 | 12 | 2 | 16.33 | 250 |
| 1 | 133 | 33 | 6 | 133 | 133 | 13 | 2 | 8.37 | 250 |
| 1 | 134 | 34 | 6 | 134 | 134 | 14 | 2 | 9.46 | 250 |
| 1 | 135 | 35 | 6 | 135 | 135 | 15 | 2 | 8.82 | 250 |
| 1 | 136 | 36 | 6 | 136 | 136 | 16 | 2 | 21.9 | 250 |
| 1 | 137 | 37 | 6 | 137 | 137 | 17 | 2 | 20.13 | 250 |
| 1 | 138 | 38 | 6 | 138 | 138 | 18 | 2 | 6.13 | 250 |
| 1 | 139 | 39 | 6 | 139 | 139 | 19 | 2 | 21.99 | 250 |
| 1 | 140 | 40 | 6 | 140 | 140 | 20 | 2 | 17.68 | 250 |
| 1 | 141 | 41 | 6 | 141 | 141 | 11 | 2 | 13.65 | 250 |
| 1 | 142 | 42 | 6 | 142 | 142 | 12 | 2 | 22.01 | 250 |
| 1 | 143 | 43 | 6 | 143 | 143 | 13 | 2 | 14.59 | 250 |
| 1 | 144 | 44 | 6 | 144 | 144 | 14 | 2 | 16.64 | 250 |
| 1 | 145 | 45 | 6 | 145 | 145 | 15 | 2 | 9.67 | 250 |
| 1 | 146 | 46 | 6 | 146 | 146 | 16 | 2 | 4.44 | 250 |
| 1 | 147 | 47 | 6 | 147 | 147 | 17 | 2 | 10.88 | 250 |
| 1 | 148 | 48 | 6 | 148 | 148 | 18 | 2 | 15.67 | 250 |
| 1 | 149 | 49 | 6 | 149 | 149 | 19 | 2 | 7.21 | 250 |
| 1 | 150 | 50 | 6 | 150 | 150 | 20 | 2 | 14.82 | 250 |
| 1 | 151 | 51 | 6 | 151 | 151 | 11 | 2 | 17.32 | 250 |
| 1 | 152 | 52 | 6 | 152 | 152 | 12 | 2 | 12.33 | 250 |
| 1 | 153 | 53 | 6 | 153 | 153 | 13 | 2 | 14.52 | 250 |
| 1 | 154 | 54 | 6 | 154 | 154 | 14 | 2 | 18.12 | 250 |
| 1 | 155 | 55 | 6 | 155 | 155 | 15 | 2 | 28.33 | 250 |
| 1 | 156 | 56 | 6 | 156 | 156 | 16 | 2 | 13.78 | 250 |
| 1 | 157 | 57 | 6 | 157 | 157 | 17 | 2 | 12.36 | 250 |
| 1 | 158 | 58 | 6 | 158 | 158 | 18 | 2 | 11.77 | 250 |
| 1 | 159 | 59 | 6 | 159 | 159 | 19 | 2 | 16.81 | 250 |
| 1 | 160 | 60 | 6 | 160 | 160 | 20 | 2 | 13.89 | 250 |
| 1 | 161 | 61 | 6 | 161 | 161 | 11 | 2 | 15.88 | 250 |
| 1 | 162 | 62 | 6 | 162 | 162 | 12 | 2 | 9.37 | 250 |
| 1 | 163 | 63 | 6 | 163 | 163 | 13 | 2 | 10.81 | 250 |
| 1 | 164 | 64 | 6 | 164 | 164 | 14 | 2 | 13.91 | 250 |
| 1 | 165 | 65 | 6 | 165 | 165 | 15 | 2 | 7.46 | 250 |
| 1 | 166 | 66 | 6 | 166 | 166 | 16 | 2 | 4.11 | 250 |
| 1 | 167 | 67 | 6 | 167 | 167 | 17 | 2 | 19.13 | 250 |
| 1 | 168 | 68 | 6 | 168 | 168 | 18 | 2 | 5.52 | 250 |
| 1 | 169 | 69 | 6 | 169 | 169 | 19 | 2 | 9.92 | 250 |
| 1 | 170 | 70 | 6 | 170 | 170 | 20 | 2 | 15.99 | 250 |
| 1 | 171 | 71 | 6 | 171 | 171 | 11 | 2 | 15.84 | 250 |
| 1 | 172 | 72 | 6 | 172 | 172 | 12 | 2 | 15.46 | 250 |
| 1 | 173 | 73 | 6 | 173 | 173 | 13 | 2 | 15.53 | 250 |
| 1 | 174 | 74 | 6 | 174 | 174 | 14 | 2 | 11.42 | 250 |
| 1 | 175 | 75 | 6 | 175 | 175 | 15 | 2 | 12.65 | 250 |
| 1 | 176 | 76 | 6 | 176 | 176 | 16 | 2 | 15.09 | 250 |
| 1 | 177 | 77 | 6 | 177 | 177 | 17 | 2 | 7.57 | 250 |
| 1 | 178 | 78 | 6 | 178 | 178 | 18 | 2 | 15.75 | 250 |
| 1 | 179 | 79 | 6 | 179 | 179 | 19 | 2 | 11.33 | 250 |
| 1 | 180 | 80 | 6 | 180 | 180 | 20 | 2 | 12.89 | 250 |
| 1 | 181 | 81 | 6 | 181 | 181 | 11 | 2 | 11.34 | 250 |
| 1 | 182 | 82 | 6 | 182 | 182 | 12 | 2 | 10 | 250 |
| 1 | 183 | 83 | 6 | 183 | 183 | 13 | 2 | 7.84 | 250 |
| 1 | 184 | 84 | 6 | 184 | 184 | 14 | 2 | 15.48 | 250 |
| 1 | 185 | 85 | 6 | 185 | 185 | 15 | 2 | 7.64 | 250 |
| 1 | 186 | 86 | 6 | 186 | 186 | 16 | 2 | 9.69 | 250 |
| 1 | 187 | 87 | 6 | 187 | 187 | 17 | 2 | 9.15 | 250 |
| 1 | 188 | 88 | 6 | 188 | 188 | 18 | 2 | 12.98 | 250 |
| 1 | 189 | 89 | 6 | 189 | 189 | 19 | 2 | 10.15 | 250 |
| 1 | 190 | 90 | 6 | 190 | 190 | 20 | 2 | 8.31 | 250 |
| 1 | 191 | 91 | 6 | 191 | 191 | 11 | 2 | 4.16 | 250 |
| 1 | 192 | 92 | 6 | 192 | 192 | 12 | 2 | 12.07 | 250 |
| 1 | 193 | 93 | 6 | 193 | 193 | 13 | 2 | 10.86 | 250 |
| 1 | 194 | 94 | 6 | 194 | 194 | 14 | 2 | 11.28 | 250 |
| 1 | 195 | 95 | 6 | 195 | 195 | 15 | 2 | 34.36 | 250 |
| 1 | 196 | 96 | 6 | 196 | 196 | 16 | 2 | 16.01 | 250 |
| 1 | 197 | 97 | 6 | 197 | 197 | 17 | 2 | 17.43 | 250 |
| 1 | 198 | 98 | 6 | 198 | 198 | 18 | 2 | 15.01 | 250 |
| 1 | 199 | 99 | 6 | 199 | 199 | 19 | 2 | 14.22 | 250 |
| 1 | 200 | 100 | 6 | 200 | 1100 | 20 | 2 | 9.84 | 250 |
| 2 | 201 | 1 | 2 | 1 | 21 | 1 | 1 | 35.21 | 500 |
| 2 | 202 | 2 | 2 | 2 | 22 | 1 | 1 | 60.21 | 500 |
| 2 | 203 | 3 | 2 | 3 | 23 | 1 | 1 | 30.2 | 500 |
| 2 | 204 | 4 | 2 | 4 | 24 | 1 | 1 | 38.32 | 500 |
| 2 | 205 | 5 | 2 | 5 | 25 | 1 | 1 | 39.66 | 500 |
| 2 | 206 | 6 | 2 | 6 | 26 | 1 | 1 | 22.63 | 500 |
| 2 | 207 | 7 | 2 | 7 | 27 | 1 | 1 | 28.75 | 500 |
| 2 | 208 | 8 | 2 | 8 | 28 | 1 | 1 | 32.08 | 500 |
| 2 | 209 | 9 | 2 | 9 | 29 | 1 | 1 | 28.54 | 500 |
| 2 | 210 | 10 | 2 | 10 | 210 | 1 | 1 | 26.98 | 500 |
| 2 | 211 | 11 | 2 | 11 | 211 | 2 | 1 | 40.38 | 500 |
| 2 | 212 | 12 | 2 | 12 | 212 | 2 | 1 | 37.64 | 500 |
| 2 | 213 | 13 | 2 | 13 | 213 | 2 | 1 | 36.62 | 500 |
| 2 | 214 | 14 | 2 | 14 | 214 | 2 | 1 | 29.35 | 500 |
| 2 | 215 | 15 | 2 | 15 | 215 | 2 | 1 | 30.83 | 500 |
| 2 | 216 | 16 | 2 | 16 | 216 | 2 | 1 | 20.28 | 500 |
| 2 | 217 | 17 | 2 | 17 | 217 | 2 | 1 | 33.86 | 500 |
| 2 | 218 | 18 | 2 | 18 | 218 | 2 | 1 | 29.43 | 500 |
| 2 | 219 | 19 | 2 | 19 | 219 | 2 | 1 | 37.28 | 500 |
| 2 | 220 | 20 | 2 | 20 | 220 | 2 | 1 | 28.18 | 500 |
| 2 | 221 | 21 | 2 | 21 | 221 | 3 | 1 | 50.41 | 500 |
| 2 | 222 | 22 | 2 | 22 | 222 | 3 | 1 | 27.04 | 500 |
| 2 | 223 | 23 | 2 | 23 | 223 | 3 | 1 | 34 | 500 |
| 2 | 224 | 24 | 2 | 24 | 224 | 3 | 1 | 37.73 | 500 |
| 2 | 225 | 25 | 2 | 25 | 225 | 3 | 1 | 19.75 | 500 |
| 2 | 226 | 26 | 2 | 26 | 226 | 3 | 1 | 38.4 | 500 |
| 2 | 227 | 27 | 2 | 27 | 227 | 3 | 1 | 37.76 | 500 |
| 2 | 228 | 28 | 2 | 28 | 228 | 3 | 1 | 13.05 | 500 |
| 2 | 229 | 29 | 2 | 29 | 229 | 3 | 1 | 32.27 | 500 |
| 2 | 230 | 30 | 2 | 30 | 230 | 3 | 1 | 25.89 | 500 |
| 2 | 231 | 31 | 2 | 31 | 231 | 4 | 1 | 36.97 | 500 |
| 2 | 232 | 32 | 2 | 32 | 232 | 4 | 1 | 28.91 | 500 |
| 2 | 233 | 33 | 2 | 33 | 233 | 4 | 1 | 25.2 | 500 |
| 2 | 234 | 34 | 2 | 34 | 234 | 4 | 1 | 23.34 | 500 |
| 2 | 235 | 35 | 2 | 35 | 235 | 4 | 1 | 23.68 | 500 |
| 2 | 236 | 36 | 2 | 36 | 236 | 4 | 1 | 27.5 | 500 |
| 2 | 237 | 37 | 2 | 37 | 237 | 4 | 1 | 32.97 | 500 |
| 2 | 238 | 38 | 2 | 38 | 238 | 4 | 1 | 33.41 | 500 |
| 2 | 239 | 39 | 2 | 39 | 239 | 4 | 1 | 35.48 | 500 |
| 2 | 240 | 40 | 2 | 40 | 240 | 4 | 1 | 32.6 | 500 |
| 2 | 241 | 41 | 2 | 41 | 241 | 5 | 1 | 45.6 | 500 |
| 2 | 242 | 42 | 2 | 42 | 242 | 5 | 1 | 28.43 | 500 |
| 2 | 243 | 43 | 2 | 43 | 243 | 5 | 1 | 12.69 | 500 |
| 2 | 244 | 44 | 2 | 44 | 244 | 5 | 1 | 32.72 | 500 |
| 2 | 245 | 45 | 2 | 45 | 245 | 5 | 1 | 27.84 | 500 |
| 2 | 246 | 46 | 2 | 46 | 246 | 5 | 1 | 19.75 | 500 |
| 2 | 247 | 47 | 2 | 47 | 247 | 5 | 1 | 37.97 | 500 |
| 2 | 248 | 48 | 2 | 48 | 248 | 5 | 1 | 19.79 | 500 |
| 2 | 249 | 49 | 2 | 49 | 249 | 5 | 1 | 6.54 | 500 |
| 2 | 250 | 50 | 2 | 50 | 250 | 5 | 1 | 30.62 | 500 |
| 2 | 251 | 51 | 2 | 51 | 251 | 6 | 1 | 46.02 | 500 |
| 2 | 252 | 52 | 2 | 52 | 252 | 6 | 1 | 17.46 | 500 |
| 2 | 253 | 53 | 2 | 53 | 253 | 6 | 1 | 49.76 | 500 |
| 2 | 254 | 54 | 2 | 54 | 254 | 6 | 1 | 31.62 | 500 |
| 2 | 255 | 55 | 2 | 55 | 255 | 6 | 1 | 58.42 | 500 |
| 2 | 256 | 56 | 2 | 56 | 256 | 6 | 1 | 20.38 | 500 |
| 2 | 257 | 57 | 2 | 57 | 257 | 6 | 1 | 44.91 | 500 |
| 2 | 258 | 58 | 2 | 58 | 258 | 6 | 1 | 24.38 | 500 |
| 2 | 259 | 59 | 2 | 59 | 259 | 6 | 1 | 36.51 | 500 |
| 2 | 260 | 60 | 2 | 60 | 260 | 6 | 1 | 39.85 | 500 |
| 2 | 261 | 61 | 2 | 61 | 261 | 7 | 1 | 31.49 | 500 |
| 2 | 262 | 62 | 2 | 62 | 262 | 7 | 1 | 35.6 | 500 |
| 2 | 263 | 63 | 2 | 63 | 263 | 7 | 1 | 27.23 | 500 |
| 2 | 264 | 64 | 2 | 64 | 264 | 7 | 1 | 32.13 | 500 |
| 2 | 265 | 65 | 2 | 65 | 265 | 7 | 1 | 22.72 | 500 |
| 2 | 266 | 66 | 2 | 66 | 266 | 7 | 1 | 23.61 | 500 |
| 2 | 267 | 67 | 2 | 67 | 267 | 7 | 1 | 21.1 | 500 |
| 2 | 268 | 68 | 2 | 68 | 268 | 7 | 1 | 21.01 | 500 |
| 2 | 269 | 69 | 2 | 69 | 269 | 7 | 1 | 41.16 | 500 |
| 2 | 270 | 70 | 2 | 70 | 270 | 7 | 1 | 37.08 | 500 |
| 2 | 271 | 71 | 2 | 71 | 271 | 8 | 1 | 24.88 | 500 |
| 2 | 272 | 72 | 2 | 72 | 272 | 8 | 1 | 34.86 | 500 |
| 2 | 273 | 73 | 2 | 73 | 273 | 8 | 1 | 18.52 | 500 |
| 2 | 274 | 74 | 2 | 74 | 274 | 8 | 1 | 19.09 | 500 |
| 2 | 275 | 75 | 2 | 75 | 275 | 8 | 1 | 41.21 | 500 |
| 2 | 276 | 76 | 2 | 76 | 276 | 8 | 1 | 24.69 | 500 |
| 2 | 277 | 77 | 2 | 77 | 277 | 8 | 1 | 13.35 | 500 |
| 2 | 278 | 78 | 2 | 78 | 278 | 8 | 1 | 34.22 | 500 |
| 2 | 279 | 79 | 2 | 79 | 279 | 8 | 1 | 32.62 | 500 |
| 2 | 280 | 80 | 2 | 80 | 280 | 8 | 1 | 27.96 | 500 |
| 2 | 281 | 81 | 2 | 81 | 281 | 9 | 1 | 28.39 | 500 |
| 2 | 282 | 82 | 2 | 82 | 282 | 9 | 1 | 40.85 | 500 |
| 2 | 283 | 83 | 2 | 83 | 283 | 9 | 1 | 13.94 | 500 |
| 2 | 284 | 84 | 2 | 84 | 284 | 9 | 1 | 32.13 | 500 |
| 2 | 285 | 85 | 2 | 85 | 285 | 9 | 1 | 26.2 | 500 |
| 2 | 286 | 86 | 2 | 86 | 286 | 9 | 1 | 34.44 | 500 |
| 2 | 287 | 87 | 2 | 87 | 287 | 9 | 1 | 18.79 | 500 |
| 2 | 288 | 88 | 2 | 88 | 288 | 9 | 1 | 28.14 | 500 |
| 2 | 289 | 89 | 2 | 89 | 289 | 9 | 1 | 30.84 | 500 |
| 2 | 290 | 90 | 2 | 90 | 290 | 9 | 1 | 19.34 | 500 |
| 2 | 291 | 91 | 2 | 91 | 291 | 10 | 1 | 20.63 | 500 |
| 2 | 292 | 92 | 2 | 92 | 292 | 10 | 1 | 21.81 | 500 |
| 2 | 293 | 93 | 2 | 93 | 293 | 10 | 1 | 32.16 | 500 |
| 2 | 294 | 94 | 2 | 94 | 294 | 10 | 1 | 9.87 | 500 |
| 2 | 295 | 95 | 2 | 95 | 295 | 10 | 1 | 64.19 | 500 |
| 2 | 296 | 96 | 2 | 96 | 296 | 10 | 1 | 34.43 | 500 |
| 2 | 297 | 97 | 2 | 97 | 297 | 10 | 1 | 27.09 | 500 |
| 2 | 298 | 98 | 2 | 98 | 298 | 10 | 1 | 24.34 | 500 |
| 2 | 299 | 99 | 2 | 99 | 299 | 10 | 1 | 30.91 | 500 |
| 2 | 300 | 100 | 2 | 100 | 2100 | 10 | 1 | 14.79 | 500 |
| 2 | 301 | 1 | 7 | 101 | 21 | 11 | 2 | 31.05 | 500 |
| 2 | 302 | 2 | 7 | 102 | 22 | 12 | 2 | 52.93 | 500 |
| 2 | 303 | 3 | 7 | 103 | 23 | 13 | 2 | 32.79 | 500 |
| 2 | 304 | 4 | 7 | 104 | 24 | 14 | 2 | 27.09 | 500 |
| 2 | 305 | 5 | 7 | 105 | 25 | 15 | 2 | 25.56 | 500 |
| 2 | 306 | 6 | 7 | 106 | 26 | 16 | 2 | 16.46 | 500 |
| 2 | 307 | 7 | 7 | 107 | 27 | 17 | 2 | 27.94 | 500 |
| 2 | 308 | 8 | 7 | 108 | 28 | 18 | 2 | 18.36 | 500 |
| 2 | 309 | 9 | 7 | 109 | 29 | 19 | 2 | 25.49 | 500 |
| 2 | 310 | 10 | 7 | 110 | 210 | 20 | 2 | 23.04 | 500 |
| 2 | 311 | 11 | 7 | 111 | 211 | 11 | 2 | 26.14 | 500 |
| 2 | 312 | 12 | 7 | 112 | 212 | 12 | 2 | 23.14 | 500 |
| 2 | 313 | 13 | 7 | 113 | 213 | 13 | 2 | 28.45 | 500 |
| 2 | 314 | 14 | 7 | 114 | 214 | 14 | 2 | 25.18 | 500 |
| 2 | 315 | 15 | 7 | 115 | 215 | 15 | 2 | 20.79 | 500 |
| 2 | 316 | 16 | 7 | 116 | 216 | 16 | 2 | 26.4 | 500 |
| 2 | 317 | 17 | 7 | 117 | 217 | 17 | 2 | 17.16 | 500 |
| 2 | 318 | 18 | 7 | 118 | 218 | 18 | 2 | 31.69 | 500 |
| 2 | 319 | 19 | 7 | 119 | 219 | 19 | 2 | 26.24 | 500 |
| 2 | 320 | 20 | 7 | 120 | 220 | 20 | 2 | 39.05 | 500 |
| 2 | 321 | 21 | 7 | 121 | 221 | 11 | 2 | 41.16 | 500 |
| 2 | 322 | 22 | 7 | 122 | 222 | 12 | 2 | 21.87 | 500 |
| 2 | 323 | 23 | 7 | 123 | 223 | 13 | 2 | 20.36 | 500 |
| 2 | 324 | 24 | 7 | 124 | 224 | 14 | 2 | 23.86 | 500 |
| 2 | 325 | 25 | 7 | 125 | 225 | 15 | 2 | 23.3 | 500 |
| 2 | 326 | 26 | 7 | 126 | 226 | 16 | 2 | 46.44 | 500 |
| 2 | 327 | 27 | 7 | 127 | 227 | 17 | 2 | 27.72 | 500 |
| 2 | 328 | 28 | 7 | 128 | 228 | 18 | 2 | 18 | 500 |
| 2 | 329 | 29 | 7 | 129 | 229 | 19 | 2 | 24.02 | 500 |
| 2 | 330 | 30 | 7 | 130 | 230 | 20 | 2 | 22.92 | 500 |
| 2 | 331 | 31 | 7 | 131 | 231 | 11 | 2 | 20.35 | 500 |
| 2 | 332 | 32 | 7 | 132 | 232 | 12 | 2 | 39.89 | 500 |
| 2 | 333 | 33 | 7 | 133 | 233 | 13 | 2 | 23.77 | 500 |
| 2 | 334 | 34 | 7 | 134 | 234 | 14 | 2 | 17.07 | 500 |
| 2 | 335 | 35 | 7 | 135 | 235 | 15 | 2 | 20.62 | 500 |
| 2 | 336 | 36 | 7 | 136 | 236 | 16 | 2 | 28.66 | 500 |
| 2 | 337 | 37 | 7 | 137 | 237 | 17 | 2 | 39.3 | 500 |
| 2 | 338 | 38 | 7 | 138 | 238 | 18 | 2 | 16.63 | 500 |
| 2 | 339 | 39 | 7 | 139 | 239 | 19 | 2 | 37.56 | 500 |
| 2 | 340 | 40 | 7 | 140 | 240 | 20 | 2 | 34.53 | 500 |
| 2 | 341 | 41 | 7 | 141 | 241 | 11 | 2 | 41.65 | 500 |
| 2 | 342 | 42 | 7 | 142 | 242 | 12 | 2 | 42.31 | 500 |
| 2 | 343 | 43 | 7 | 143 | 243 | 13 | 2 | 32.31 | 500 |
| 2 | 344 | 44 | 7 | 144 | 244 | 14 | 2 | 37.06 | 500 |
| 2 | 345 | 45 | 7 | 145 | 245 | 15 | 2 | 15.01 | 500 |
| 2 | 346 | 46 | 7 | 146 | 246 | 16 | 2 | 11.46 | 500 |
| 2 | 347 | 47 | 7 | 147 | 247 | 17 | 2 | 30.32 | 500 |
| 2 | 348 | 48 | 7 | 148 | 248 | 18 | 2 | 34.52 | 500 |
| 2 | 349 | 49 | 7 | 149 | 249 | 19 | 2 | 8.54 | 500 |
| 2 | 350 | 50 | 7 | 150 | 250 | 20 | 2 | 31.94 | 500 |
| 2 | 351 | 51 | 7 | 151 | 251 | 11 | 2 | 51.28 | 500 |
| 2 | 352 | 52 | 7 | 152 | 252 | 12 | 2 | 36.95 | 500 |
| 2 | 353 | 53 | 7 | 153 | 253 | 13 | 2 | 33.79 | 500 |
| 2 | 354 | 54 | 7 | 154 | 254 | 14 | 2 | 44.58 | 500 |
| 2 | 355 | 55 | 7 | 155 | 255 | 15 | 2 | 40.24 | 500 |
| 2 | 356 | 56 | 7 | 156 | 256 | 16 | 2 | 30.63 | 500 |
| 2 | 357 | 57 | 7 | 157 | 257 | 17 | 2 | 36.39 | 500 |
| 2 | 358 | 58 | 7 | 158 | 258 | 18 | 2 | 24.97 | 500 |
| 2 | 359 | 59 | 7 | 159 | 259 | 19 | 2 | 32.44 | 500 |
| 2 | 360 | 60 | 7 | 160 | 260 | 20 | 2 | 29.19 | 500 |
| 2 | 361 | 61 | 7 | 161 | 261 | 11 | 2 | 42.8 | 500 |
| 2 | 362 | 62 | 7 | 162 | 262 | 12 | 2 | 19.76 | 500 |
| 2 | 363 | 63 | 7 | 163 | 263 | 13 | 2 | 24.87 | 500 |
| 2 | 364 | 64 | 7 | 164 | 264 | 14 | 2 | 41.63 | 500 |
| 2 | 365 | 65 | 7 | 165 | 265 | 15 | 2 | 19.05 | 500 |
| 2 | 366 | 66 | 7 | 166 | 266 | 16 | 2 | 16.49 | 500 |
| 2 | 367 | 67 | 7 | 167 | 267 | 17 | 2 | 32.87 | 500 |
| 2 | 368 | 68 | 7 | 168 | 268 | 18 | 2 | 18.11 | 500 |
| 2 | 369 | 69 | 7 | 169 | 269 | 19 | 2 | 27.83 | 500 |
| 2 | 370 | 70 | 7 | 170 | 270 | 20 | 2 | 42.19 | 500 |
| 2 | 371 | 71 | 7 | 171 | 271 | 11 | 2 | 40.52 | 500 |
| 2 | 372 | 72 | 7 | 172 | 272 | 12 | 2 | 36.27 | 500 |
| 2 | 373 | 73 | 7 | 173 | 273 | 13 | 2 | 28.33 | 500 |
| 2 | 374 | 74 | 7 | 174 | 274 | 14 | 2 | 23.28 | 500 |
| 2 | 375 | 75 | 7 | 175 | 275 | 15 | 2 | 30.22 | 500 |
| 2 | 376 | 76 | 7 | 176 | 276 | 16 | 2 | 33.02 | 500 |
| 2 | 377 | 77 | 7 | 177 | 277 | 17 | 2 | 17.04 | 500 |
| 2 | 378 | 78 | 7 | 178 | 278 | 18 | 2 | 43.64 | 500 |
| 2 | 379 | 79 | 7 | 179 | 279 | 19 | 2 | 31.91 | 500 |
| 2 | 380 | 80 | 7 | 180 | 280 | 20 | 2 | 41.45 | 500 |
| 2 | 381 | 81 | 7 | 181 | 281 | 11 | 2 | 44.79 | 500 |
| 2 | 382 | 82 | 7 | 182 | 282 | 12 | 2 | 24.26 | 500 |
| 2 | 383 | 83 | 7 | 183 | 283 | 13 | 2 | 25.53 | 500 |
| 2 | 384 | 84 | 7 | 184 | 284 | 14 | 2 | 37.89 | 500 |
| 2 | 385 | 85 | 7 | 185 | 285 | 15 | 2 | 20.24 | 500 |
| 2 | 386 | 86 | 7 | 186 | 286 | 16 | 2 | 32.39 | 500 |
| 2 | 387 | 87 | 7 | 187 | 287 | 17 | 2 | 22.14 | 500 |
| 2 | 388 | 88 | 7 | 188 | 288 | 18 | 2 | 37.16 | 500 |
| 2 | 389 | 89 | 7 | 189 | 289 | 19 | 2 | 28.99 | 500 |
| 2 | 390 | 90 | 7 | 190 | 290 | 20 | 2 | 22.1 | 500 |
| 2 | 391 | 91 | 7 | 191 | 291 | 11 | 2 | 8.28 | 500 |
| 2 | 392 | 92 | 7 | 192 | 292 | 12 | 2 | 29.71 | 500 |
| 2 | 393 | 93 | 7 | 193 | 293 | 13 | 2 | 36.75 | 500 |
| 2 | 394 | 94 | 7 | 194 | 294 | 14 | 2 | 18.82 | 500 |
| 2 | 395 | 95 | 7 | 195 | 295 | 15 | 2 | 61.41 | 500 |
| 2 | 396 | 96 | 7 | 196 | 296 | 16 | 2 | 29.89 | 500 |
| 2 | 397 | 97 | 7 | 197 | 297 | 17 | 2 | 35.74 | 500 |
| 2 | 398 | 98 | 7 | 198 | 298 | 18 | 2 | 23.78 | 500 |
| 2 | 399 | 99 | 7 | 199 | 299 | 19 | 2 | 19.14 | 500 |
| 2 | 400 | 100 | 7 | 200 | 2100 | 20 | 2 | 15.75 | 500 |
| 3 | 401 | 1 | 3 | 1 | 31 | 1 | 1 | 30.63 | 815 |
| 3 | 402 | 2 | 3 | 2 | 32 | 1 | 1 | 45.92 | 815 |
| 3 | 403 | 3 | 3 | 3 | 33 | 1 | 1 | 29.95 | 815 |
| 3 | 404 | 4 | 3 | 4 | 34 | 1 | 1 | 31.54 | 815 |
| 3 | 405 | 5 | 3 | 5 | 35 | 1 | 1 | 21.46 | 815 |
| 3 | 406 | 6 | 3 | 6 | 36 | 1 | 1 | 11.25 | 815 |
| 3 | 407 | 7 | 3 | 7 | 37 | 1 | 1 | 32.04 | 815 |
| 3 | 408 | 8 | 3 | 8 | 38 | 1 | 1 | 11.69 | 815 |
| 3 | 409 | 9 | 3 | 9 | 39 | 1 | 1 | 31.9 | 815 |
| 3 | 410 | 10 | 3 | 10 | 310 | 1 | 1 | 23.37 | 815 |
| 3 | 411 | 11 | 3 | 11 | 311 | 2 | 1 | 21.14 | 815 |
| 3 | 412 | 12 | 3 | 12 | 312 | 2 | 1 | 20.36 | 815 |
| 3 | 413 | 13 | 3 | 13 | 313 | 2 | 1 | 14.72 | 815 |
| 3 | 414 | 14 | 3 | 14 | 314 | 2 | 1 | 14.98 | 815 |
| 3 | 415 | 15 | 3 | 15 | 315 | 2 | 1 | 14.81 | 815 |
| 3 | 416 | 16 | 3 | 16 | 316 | 2 | 1 | 15.27 | 815 |
| 3 | 417 | 17 | 3 | 17 | 317 | 2 | 1 | 15.54 | 815 |
| 3 | 418 | 18 | 3 | 18 | 318 | 2 | 1 | 31.43 | 815 |
| 3 | 419 | 19 | 3 | 19 | 319 | 2 | 1 | 26.3 | 815 |
| 3 | 420 | 20 | 3 | 20 | 320 | 2 | 1 | 14.57 | 815 |
| 3 | 421 | 21 | 3 | 21 | 321 | 3 | 1 | 32.88 | 815 |
| 3 | 422 | 22 | 3 | 22 | 322 | 3 | 1 | 24.01 | 815 |
| 3 | 423 | 23 | 3 | 23 | 323 | 3 | 1 | 14.13 | 815 |
| 3 | 424 | 24 | 3 | 24 | 324 | 3 | 1 | 23.75 | 815 |
| 3 | 425 | 25 | 3 | 25 | 325 | 3 | 1 | 13.05 | 815 |
| 3 | 426 | 26 | 3 | 26 | 326 | 3 | 1 | 20.51 | 815 |
| 3 | 427 | 27 | 3 | 27 | 327 | 3 | 1 | 22.02 | 815 |
| 3 | 428 | 28 | 3 | 28 | 328 | 3 | 1 | 10.23 | 815 |
| 3 | 429 | 29 | 3 | 29 | 329 | 3 | 1 | 24.25 | 815 |
| 3 | 430 | 30 | 3 | 30 | 330 | 3 | 1 | 8.73 | 815 |
| 3 | 431 | 31 | 3 | 31 | 331 | 4 | 1 | 17.09 | 815 |
| 3 | 432 | 32 | 3 | 32 | 332 | 4 | 1 | 12.71 | 815 |
| 3 | 433 | 33 | 3 | 33 | 333 | 4 | 1 | 23.44 | 815 |
| 3 | 434 | 34 | 3 | 34 | 334 | 4 | 1 | 10.19 | 815 |
| 3 | 435 | 35 | 3 | 35 | 335 | 4 | 1 | 10.46 | 815 |
| 3 | 436 | 36 | 3 | 36 | 336 | 4 | 1 | 19.45 | 815 |
| 3 | 437 | 37 | 3 | 37 | 337 | 4 | 1 | 19.02 | 815 |
| 3 | 438 | 38 | 3 | 38 | 338 | 4 | 1 | 17.72 | 815 |
| 3 | 439 | 39 | 3 | 39 | 339 | 4 | 1 | 17.73 | 815 |
| 3 | 440 | 40 | 3 | 40 | 340 | 4 | 1 | 19.41 | 815 |
| 3 | 441 | 41 | 3 | 41 | 341 | 5 | 1 | 25.83 | 815 |
| 3 | 442 | 42 | 3 | 42 | 342 | 5 | 1 | 21.55 | 815 |
| 3 | 443 | 43 | 3 | 43 | 343 | 5 | 1 | 19.45 | 815 |
| 3 | 444 | 44 | 3 | 44 | 344 | 5 | 1 | 41.69 | 815 |
| 3 | 445 | 45 | 3 | 45 | 345 | 5 | 1 | 19.28 | 815 |
| 3 | 446 | 46 | 3 | 46 | 346 | 5 | 1 | 14.53 | 815 |
| 3 | 447 | 47 | 3 | 47 | 347 | 5 | 1 | 30.47 | 815 |
| 3 | 448 | 48 | 3 | 48 | 348 | 5 | 1 | 16.48 | 815 |
| 3 | 449 | 49 | 3 | 49 | 349 | 5 | 1 | 5.87 | 815 |
| 3 | 450 | 50 | 3 | 50 | 350 | 5 | 1 | 27.56 | 815 |
| 3 | 451 | 51 | 3 | 51 | 351 | 6 | 1 | 32.06 | 815 |
| 3 | 452 | 52 | 3 | 52 | 352 | 6 | 1 | 18.22 | 815 |
| 3 | 453 | 53 | 3 | 53 | 353 | 6 | 1 | 22.32 | 815 |
| 3 | 454 | 54 | 3 | 54 | 354 | 6 | 1 | 23.98 | 815 |
| 3 | 455 | 55 | 3 | 55 | 355 | 6 | 1 | 26.39 | 815 |
| 3 | 456 | 56 | 3 | 56 | 356 | 6 | 1 | 8.52 | 815 |
| 3 | 457 | 57 | 3 | 57 | 357 | 6 | 1 | 21.5 | 815 |
| 3 | 458 | 58 | 3 | 58 | 358 | 6 | 1 | 12.5 | 815 |
| 3 | 459 | 59 | 3 | 59 | 359 | 6 | 1 | 21.98 | 815 |
| 3 | 460 | 60 | 3 | 60 | 360 | 6 | 1 | 18.32 | 815 |
| 3 | 461 | 61 | 3 | 61 | 361 | 7 | 1 | 16.12 | 815 |
| 3 | 462 | 62 | 3 | 62 | 362 | 7 | 1 | 21.69 | 815 |
| 3 | 463 | 63 | 3 | 63 | 363 | 7 | 1 | 20.39 | 815 |
| 3 | 464 | 64 | 3 | 64 | 364 | 7 | 1 | 15.11 | 815 |
| 3 | 465 | 65 | 3 | 65 | 365 | 7 | 1 | 12.28 | 815 |
| 3 | 466 | 66 | 3 | 66 | 366 | 7 | 1 | 20.89 | 815 |
| 3 | 467 | 67 | 3 | 67 | 367 | 7 | 1 | 19.64 | 815 |
| 3 | 468 | 68 | 3 | 68 | 368 | 7 | 1 | 12.69 | 815 |
| 3 | 469 | 69 | 3 | 69 | 369 | 7 | 1 | 13.42 | 815 |
| 3 | 470 | 70 | 3 | 70 | 370 | 7 | 1 | 33.63 | 815 |
| 3 | 471 | 71 | 3 | 71 | 371 | 8 | 1 | 9.89 | 815 |
| 3 | 472 | 72 | 3 | 72 | 372 | 8 | 1 | 34.37 | 815 |
| 3 | 473 | 73 | 3 | 73 | 373 | 8 | 1 | 37.36 | 815 |
| 3 | 474 | 74 | 3 | 74 | 374 | 8 | 1 | 9.47 | 815 |
| 3 | 475 | 75 | 3 | 75 | 375 | 8 | 1 | 32.46 | 815 |
| 3 | 476 | 76 | 3 | 76 | 376 | 8 | 1 | 17.67 | 815 |
| 3 | 477 | 77 | 3 | 77 | 377 | 8 | 1 | 9.65 | 815 |
| 3 | 478 | 78 | 3 | 78 | 378 | 8 | 1 | 21.7 | 815 |
| 3 | 479 | 79 | 3 | 79 | 379 | 8 | 1 | 14.99 | 815 |
| 3 | 480 | 80 | 3 | 80 | 380 | 8 | 1 | 26.86 | 815 |
| 3 | 481 | 81 | 3 | 81 | 381 | 9 | 1 | 13.87 | 815 |
| 3 | 482 | 82 | 3 | 82 | 382 | 9 | 1 | 27.11 | 815 |
| 3 | 483 | 83 | 3 | 83 | 383 | 9 | 1 | 21.31 | 815 |
| 3 | 484 | 84 | 3 | 84 | 384 | 9 | 1 | 24.26 | 815 |
| 3 | 485 | 85 | 3 | 85 | 385 | 9 | 1 | 19.62 | 815 |
| 3 | 486 | 86 | 3 | 86 | 386 | 9 | 1 | 23.51 | 815 |
| 3 | 487 | 87 | 3 | 87 | 387 | 9 | 1 | 17.66 | 815 |
| 3 | 488 | 88 | 3 | 88 | 388 | 9 | 1 | 17.76 | 815 |
| 3 | 489 | 89 | 3 | 89 | 389 | 9 | 1 | 20.97 | 815 |
| 3 | 490 | 90 | 3 | 90 | 390 | 9 | 1 | 11.75 | 815 |
| 3 | 491 | 91 | 3 | 91 | 391 | 10 | 1 | 9.13 | 815 |
| 3 | 492 | 92 | 3 | 92 | 392 | 10 | 1 | 13.34 | 815 |
| 3 | 493 | 93 | 3 | 93 | 393 | 10 | 1 | 20.48 | 815 |
| 3 | 494 | 94 | 3 | 94 | 394 | 10 | 1 | 10.39 | 815 |
| 3 | 495 | 95 | 3 | 95 | 395 | 10 | 1 | 36.4 | 815 |
| 3 | 496 | 96 | 3 | 96 | 396 | 10 | 1 | 30.25 | 815 |
| 3 | 497 | 97 | 3 | 97 | 397 | 10 | 1 | 19.44 | 815 |
| 3 | 498 | 98 | 3 | 98 | 398 | 10 | 1 | 18.15 | 815 |
| 3 | 499 | 99 | 3 | 99 | 399 | 10 | 1 | 7.72 | 815 |
| 3 | 500 | 100 | 3 | 100 | 3100 | 10 | 1 | 7 | 815 |
| 3 | 501 | 1 | 8 | 101 | 31 | 11 | 2 | 26.95 | 815 |
| 3 | 502 | 2 | 8 | 102 | 32 | 12 | 2 | 35.14 | 815 |
| 3 | 503 | 3 | 8 | 103 | 33 | 13 | 2 | 18.66 | 815 |
| 3 | 504 | 4 | 8 | 104 | 34 | 14 | 2 | 21.98 | 815 |
| 3 | 505 | 5 | 8 | 105 | 35 | 15 | 2 | 18.75 | 815 |
| 3 | 506 | 6 | 8 | 106 | 36 | 16 | 2 | 14.05 | 815 |
| 3 | 507 | 7 | 8 | 107 | 37 | 17 | 2 | 20.42 | 815 |
| 3 | 508 | 8 | 8 | 108 | 38 | 18 | 2 | 13.24 | 815 |
| 3 | 509 | 9 | 8 | 109 | 39 | 19 | 2 | 21.03 | 815 |
| 3 | 510 | 10 | 8 | 110 | 310 | 20 | 2 | 20.01 | 815 |
| 3 | 511 | 11 | 8 | 111 | 311 | 11 | 2 | 17.76 | 815 |
| 3 | 512 | 12 | 8 | 112 | 312 | 12 | 2 | 15.38 | 815 |
| 3 | 513 | 13 | 8 | 113 | 313 | 13 | 2 | 14.63 | 815 |
| 3 | 514 | 14 | 8 | 114 | 314 | 14 | 2 | 14.79 | 815 |
| 3 | 515 | 15 | 8 | 115 | 315 | 15 | 2 | 11.19 | 815 |
| 3 | 516 | 16 | 8 | 116 | 316 | 16 | 2 | 18.76 | 815 |
| 3 | 517 | 17 | 8 | 117 | 317 | 17 | 2 | 10.13 | 815 |
| 3 | 518 | 18 | 8 | 118 | 318 | 18 | 2 | 21.08 | 815 |
| 3 | 519 | 19 | 8 | 119 | 319 | 19 | 2 | 15.8 | 815 |
| 3 | 520 | 20 | 8 | 120 | 320 | 20 | 2 | 18.88 | 815 |
| 3 | 521 | 21 | 8 | 121 | 321 | 11 | 2 | 27.71 | 815 |
| 3 | 522 | 22 | 8 | 122 | 322 | 12 | 2 | 16.91 | 815 |
| 3 | 523 | 23 | 8 | 123 | 323 | 13 | 2 | 13.46 | 815 |
| 3 | 524 | 24 | 8 | 124 | 324 | 14 | 2 | 14.59 | 815 |
| 3 | 525 | 25 | 8 | 125 | 325 | 15 | 2 | 18.42 | 815 |
| 3 | 526 | 26 | 8 | 126 | 326 | 16 | 2 | 13.93 | 815 |
| 3 | 527 | 27 | 8 | 127 | 327 | 17 | 2 | 17.33 | 815 |
| 3 | 528 | 28 | 8 | 128 | 328 | 18 | 2 | 14.91 | 815 |
| 3 | 529 | 29 | 8 | 129 | 329 | 19 | 2 | 29.7 | 815 |
| 3 | 530 | 30 | 8 | 130 | 330 | 20 | 2 | 10.56 | 815 |
| 3 | 531 | 31 | 8 | 131 | 331 | 11 | 2 | 11.85 | 815 |
| 3 | 532 | 32 | 8 | 132 | 332 | 12 | 2 | 23.55 | 815 |
| 3 | 533 | 33 | 8 | 133 | 333 | 13 | 2 | 20.13 | 815 |
| 3 | 534 | 34 | 8 | 134 | 334 | 14 | 2 | 10.09 | 815 |
| 3 | 535 | 35 | 8 | 135 | 335 | 15 | 2 | 10.27 | 815 |
| 3 | 536 | 36 | 8 | 136 | 336 | 16 | 2 | 23.46 | 815 |
| 3 | 537 | 37 | 8 | 137 | 337 | 17 | 2 | 21.44 | 815 |
| 3 | 538 | 38 | 8 | 138 | 338 | 18 | 2 | 12.89 | 815 |
| 3 | 539 | 39 | 8 | 139 | 339 | 19 | 2 | 20.18 | 815 |
| 3 | 540 | 40 | 8 | 140 | 340 | 20 | 2 | 20.56 | 815 |
| 3 | 541 | 41 | 8 | 141 | 341 | 11 | 2 | 31.69 | 815 |
| 3 | 542 | 42 | 8 | 142 | 342 | 12 | 2 | 20.8 | 815 |
| 3 | 543 | 43 | 8 | 143 | 343 | 13 | 2 | 16.3 | 815 |
| 3 | 544 | 44 | 8 | 144 | 344 | 14 | 2 | 31.81 | 815 |
| 3 | 545 | 45 | 8 | 145 | 345 | 15 | 2 | 13.49 | 815 |
| 3 | 546 | 46 | 8 | 146 | 346 | 16 | 2 | 6.68 | 815 |
| 3 | 547 | 47 | 8 | 147 | 347 | 17 | 2 | 34.18 | 815 |
| 3 | 548 | 48 | 8 | 148 | 348 | 18 | 2 | 23.08 | 815 |
| 3 | 549 | 49 | 8 | 149 | 349 | 19 | 2 | 4.41 | 815 |
| 3 | 550 | 50 | 8 | 150 | 350 | 20 | 2 | 19.85 | 815 |
| 3 | 551 | 51 | 8 | 151 | 351 | 11 | 2 | 24.73 | 815 |
| 3 | 552 | 52 | 8 | 152 | 352 | 12 | 2 | 24.77 | 815 |
| 3 | 553 | 53 | 8 | 153 | 353 | 13 | 2 | 33.65 | 815 |
| 3 | 554 | 54 | 8 | 154 | 354 | 14 | 2 | 31.21 | 815 |
| 3 | 555 | 55 | 8 | 155 | 355 | 15 | 2 | 36.07 | 815 |
| 3 | 556 | 56 | 8 | 156 | 356 | 16 | 2 | 13.51 | 815 |
| 3 | 557 | 57 | 8 | 157 | 357 | 17 | 2 | 19.91 | 815 |
| 3 | 558 | 58 | 8 | 158 | 358 | 18 | 2 | 18.94 | 815 |
| 3 | 559 | 59 | 8 | 159 | 359 | 19 | 2 | 23.46 | 815 |
| 3 | 560 | 60 | 8 | 160 | 360 | 20 | 2 | 15.86 | 815 |
| 3 | 561 | 61 | 8 | 161 | 361 | 11 | 2 | 12.51 | 815 |
| 3 | 562 | 62 | 8 | 162 | 362 | 12 | 2 | 10.35 | 815 |
| 3 | 563 | 63 | 8 | 163 | 363 | 13 | 2 | 18.02 | 815 |
| 3 | 564 | 64 | 8 | 164 | 364 | 14 | 2 | 17.77 | 815 |
| 3 | 565 | 65 | 8 | 165 | 365 | 15 | 2 | 11.78 | 815 |
| 3 | 566 | 66 | 8 | 166 | 366 | 16 | 2 | 10.72 | 815 |
| 3 | 567 | 67 | 8 | 167 | 367 | 17 | 2 | 19.72 | 815 |
| 3 | 568 | 68 | 8 | 168 | 368 | 18 | 2 | 12.65 | 815 |
| 3 | 569 | 69 | 8 | 169 | 369 | 19 | 2 | 24.75 | 815 |
| 3 | 570 | 70 | 8 | 170 | 370 | 20 | 2 | 25.65 | 815 |
| 3 | 571 | 71 | 8 | 171 | 371 | 11 | 2 | 15.28 | 815 |
| 3 | 572 | 72 | 8 | 172 | 372 | 12 | 2 | 28.5 | 815 |
| 3 | 573 | 73 | 8 | 173 | 373 | 13 | 2 | 25.63 | 815 |
| 3 | 574 | 74 | 8 | 174 | 374 | 14 | 2 | 12.6 | 815 |
| 3 | 575 | 75 | 8 | 175 | 375 | 15 | 2 | 21.47 | 815 |
| 3 | 576 | 76 | 8 | 176 | 376 | 16 | 2 | 23.4 | 815 |
| 3 | 577 | 77 | 8 | 177 | 377 | 17 | 2 | 18.6 | 815 |
| 3 | 578 | 78 | 8 | 178 | 378 | 18 | 2 | 24.35 | 815 |
| 3 | 579 | 79 | 8 | 179 | 379 | 19 | 2 | 25.3 | 815 |
| 3 | 580 | 80 | 8 | 180 | 380 | 20 | 2 | 22.4 | 815 |
| 3 | 581 | 81 | 8 | 181 | 381 | 11 | 2 | 20.55 | 815 |
| 3 | 582 | 82 | 8 | 182 | 382 | 12 | 2 | 20.03 | 815 |
| 3 | 583 | 83 | 8 | 183 | 383 | 13 | 2 | 26.26 | 815 |
| 3 | 584 | 84 | 8 | 184 | 384 | 14 | 2 | 26.58 | 815 |
| 3 | 585 | 85 | 8 | 185 | 385 | 15 | 2 | 14.13 | 815 |
| 3 | 586 | 86 | 8 | 186 | 386 | 16 | 2 | 17.43 | 815 |
| 3 | 587 | 87 | 8 | 187 | 387 | 17 | 2 | 9.35 | 815 |
| 3 | 588 | 88 | 8 | 188 | 388 | 18 | 2 | 25.99 | 815 |
| 3 | 589 | 89 | 8 | 189 | 389 | 19 | 2 | 30.33 | 815 |
| 3 | 590 | 90 | 8 | 190 | 390 | 20 | 2 | 19.01 | 815 |
| 3 | 591 | 91 | 8 | 191 | 391 | 11 | 2 | 10.64 | 815 |
| 3 | 592 | 92 | 8 | 192 | 392 | 12 | 2 | 21.66 | 815 |
| 3 | 593 | 93 | 8 | 193 | 393 | 13 | 2 | 31.33 | 815 |
| 3 | 594 | 94 | 8 | 194 | 394 | 14 | 2 | 15.25 | 815 |
| 3 | 595 | 95 | 8 | 195 | 395 | 15 | 2 | 26.97 | 815 |
| 3 | 596 | 96 | 8 | 196 | 396 | 16 | 2 | 20.94 | 815 |
| 3 | 597 | 97 | 8 | 197 | 397 | 17 | 2 | 21.47 | 815 |
| 3 | 598 | 98 | 8 | 198 | 398 | 18 | 2 | 21.47 | 815 |
| 3 | 599 | 99 | 8 | 199 | 399 | 19 | 2 | 7 | 815 |
| 3 | 600 | 100 | 8 | 200 | 3100 | 20 | 2 | 5.84 | 815 |
| 4 | 601 | 1 | 4 | 1 | 41 | 1 | 1 | 23.18 | 1405 |
| 4 | 602 | 2 | 4 | 2 | 42 | 1 | 1 | 34.09 | 1405 |
| 4 | 603 | 3 | 4 | 3 | 43 | 1 | 1 | 27.14 | 1405 |
| 4 | 604 | 4 | 4 | 4 | 44 | 1 | 1 | 17.24 | 1405 |
| 4 | 605 | 5 | 4 | 5 | 45 | 1 | 1 | 19.04 | 1405 |
| 4 | 606 | 6 | 4 | 6 | 46 | 1 | 1 | 3.31 | 1405 |
| 4 | 607 | 7 | 4 | 7 | 47 | 1 | 1 | 21.83 | 1405 |
| 4 | 608 | 8 | 4 | 8 | 48 | 1 | 1 | 5.65 | 1405 |
| 4 | 609 | 9 | 4 | 9 | 49 | 1 | 1 | 9.52 | 1405 |
| 4 | 610 | 10 | 4 | 10 | 410 | 1 | 1 | 13.61 | 1405 |
| 4 | 611 | 11 | 4 | 11 | 411 | 2 | 1 | 25 | 1405 |
| 4 | 612 | 12 | 4 | 12 | 412 | 2 | 1 | 22.2 | 1405 |
| 4 | 613 | 13 | 4 | 13 | 413 | 2 | 1 | 16.38 | 1405 |
| 4 | 614 | 14 | 4 | 14 | 414 | 2 | 1 | 12.98 | 1405 |
| 4 | 615 | 15 | 4 | 15 | 415 | 2 | 1 | 9.32 | 1405 |
| 4 | 616 | 16 | 4 | 16 | 416 | 2 | 1 | 13.63 | 1405 |
| 4 | 617 | 17 | 4 | 17 | 417 | 2 | 1 | 12.67 | 1405 |
| 4 | 618 | 18 | 4 | 18 | 418 | 2 | 1 | 25.93 | 1405 |
| 4 | 619 | 19 | 4 | 19 | 419 | 2 | 1 | 19.58 | 1405 |
| 4 | 620 | 20 | 4 | 20 | 420 | 2 | 1 | 12.76 | 1405 |
| 4 | 621 | 21 | 4 | 21 | 421 | 3 | 1 | 33.66 | 1405 |
| 4 | 622 | 22 | 4 | 22 | 422 | 3 | 1 | 16.29 | 1405 |
| 4 | 623 | 23 | 4 | 23 | 423 | 3 | 1 | 10.24 | 1405 |
| 4 | 624 | 24 | 4 | 24 | 424 | 3 | 1 | 15.44 | 1405 |
| 4 | 625 | 25 | 4 | 25 | 425 | 3 | 1 | 13.18 | 1405 |
| 4 | 626 | 26 | 4 | 26 | 426 | 3 | 1 | 22.66 | 1405 |
| 4 | 627 | 27 | 4 | 27 | 427 | 3 | 1 | 26.08 | 1405 |
| 4 | 628 | 28 | 4 | 28 | 428 | 3 | 1 | 3.53 | 1405 |
| 4 | 629 | 29 | 4 | 29 | 429 | 3 | 1 | 25.69 | 1405 |
| 4 | 630 | 30 | 4 | 30 | 430 | 3 | 1 | 4.42 | 1405 |
| 4 | 631 | 31 | 4 | 31 | 431 | 4 | 1 | 13.64 | 1405 |
| 4 | 632 | 32 | 4 | 32 | 432 | 4 | 1 | 15.1 | 1405 |
| 4 | 633 | 33 | 4 | 33 | 433 | 4 | 1 | 23.28 | 1405 |
| 4 | 634 | 34 | 4 | 34 | 434 | 4 | 1 | 8.01 | 1405 |
| 4 | 635 | 35 | 4 | 35 | 435 | 4 | 1 | 6.02 | 1405 |
| 4 | 636 | 36 | 4 | 36 | 436 | 4 | 1 | 14.42 | 1405 |
| 4 | 637 | 37 | 4 | 37 | 437 | 4 | 1 | 13.61 | 1405 |
| 4 | 638 | 38 | 4 | 38 | 438 | 4 | 1 | 15.15 | 1405 |
| 4 | 639 | 39 | 4 | 39 | 439 | 4 | 1 | 8.24 | 1405 |
| 4 | 640 | 40 | 4 | 40 | 440 | 4 | 1 | 24.51 | 1405 |
| 4 | 641 | 41 | 4 | 41 | 441 | 5 | 1 | 24.4 | 1405 |
| 4 | 642 | 42 | 4 | 42 | 442 | 5 | 1 | 20.86 | 1405 |
| 4 | 643 | 43 | 4 | 43 | 443 | 5 | 1 | 28.28 | 1405 |
| 4 | 644 | 44 | 4 | 44 | 444 | 5 | 1 | 40.04 | 1405 |
| 4 | 645 | 45 | 4 | 45 | 445 | 5 | 1 | 13.64 | 1405 |
| 4 | 646 | 46 | 4 | 46 | 446 | 5 | 1 | 7.2 | 1405 |
| 4 | 647 | 47 | 4 | 47 | 447 | 5 | 1 | 36.59 | 1405 |
| 4 | 648 | 48 | 4 | 48 | 448 | 5 | 1 | 6.36 | 1405 |
| 4 | 649 | 49 | 4 | 49 | 449 | 5 | 1 | NA | 1405 |
| 4 | 650 | 50 | 4 | 50 | 450 | 5 | 1 | 17.1 | 1405 |
| 4 | 651 | 51 | 4 | 51 | 451 | 6 | 1 | 16.22 | 1405 |
| 4 | 652 | 52 | 4 | 52 | 452 | 6 | 1 | 8.24 | 1405 |
| 4 | 653 | 53 | 4 | 53 | 453 | 6 | 1 | 20.05 | 1405 |
| 4 | 654 | 54 | 4 | 54 | 454 | 6 | 1 | 15.28 | 1405 |
| 4 | 655 | 55 | 4 | 55 | 455 | 6 | 1 | 17.47 | 1405 |
| 4 | 656 | 56 | 4 | 56 | 456 | 6 | 1 | 3.58 | 1405 |
| 4 | 657 | 57 | 4 | 57 | 457 | 6 | 1 | 17.58 | 1405 |
| 4 | 658 | 58 | 4 | 58 | 458 | 6 | 1 | 13.55 | 1405 |
| 4 | 659 | 59 | 4 | 59 | 459 | 6 | 1 | 11.33 | 1405 |
| 4 | 660 | 60 | 4 | 60 | 460 | 6 | 1 | 10.5 | 1405 |
| 4 | 661 | 61 | 4 | 61 | 461 | 7 | 1 | 17.29 | 1405 |
| 4 | 662 | 62 | 4 | 62 | 462 | 7 | 1 | 11.33 | 1405 |
| 4 | 663 | 63 | 4 | 63 | 463 | 7 | 1 | 18.4 | 1405 |
| 4 | 664 | 64 | 4 | 64 | 464 | 7 | 1 | 10.86 | 1405 |
| 4 | 665 | 65 | 4 | 65 | 465 | 7 | 1 | 10.51 | 1405 |
| 4 | 666 | 66 | 4 | 66 | 466 | 7 | 1 | 14.36 | 1405 |
| 4 | 667 | 67 | 4 | 67 | 467 | 7 | 1 | 7.1 | 1405 |
| 4 | 668 | 68 | 4 | 68 | 468 | 7 | 1 | 14.26 | 1405 |
| 4 | 669 | 69 | 4 | 69 | 469 | 7 | 1 | 8.81 | 1405 |
| 4 | 670 | 70 | 4 | 70 | 470 | 7 | 1 | 30.81 | 1405 |
| 4 | 671 | 71 | 4 | 71 | 471 | 8 | 1 | 9.27 | 1405 |
| 4 | 672 | 72 | 4 | 72 | 472 | 8 | 1 | 36.87 | 1405 |
| 4 | 673 | 73 | 4 | 73 | 473 | 8 | 1 | 24.8 | 1405 |
| 4 | 674 | 74 | 4 | 74 | 474 | 8 | 1 | 1.91 | 1405 |
| 4 | 675 | 75 | 4 | 75 | 475 | 8 | 1 | 30.17 | 1405 |
| 4 | 676 | 76 | 4 | 76 | 476 | 8 | 1 | 18.35 | 1405 |
| 4 | 677 | 77 | 4 | 77 | 477 | 8 | 1 | 2.37 | 1405 |
| 4 | 678 | 78 | 4 | 78 | 478 | 8 | 1 | 20.59 | 1405 |
| 4 | 679 | 79 | 4 | 79 | 479 | 8 | 1 | 13.79 | 1405 |
| 4 | 680 | 80 | 4 | 80 | 480 | 8 | 1 | 15.23 | 1405 |
| 4 | 681 | 81 | 4 | 81 | 481 | 9 | 1 | 12.23 | 1405 |
| 4 | 682 | 82 | 4 | 82 | 482 | 9 | 1 | 24.35 | 1405 |
| 4 | 683 | 83 | 4 | 83 | 483 | 9 | 1 | 10.7 | 1405 |
| 4 | 684 | 84 | 4 | 84 | 484 | 9 | 1 | 14.29 | 1405 |
| 4 | 685 | 85 | 4 | 85 | 485 | 9 | 1 | 15.51 | 1405 |
| 4 | 686 | 86 | 4 | 86 | 486 | 9 | 1 | 16.37 | 1405 |
| 4 | 687 | 87 | 4 | 87 | 487 | 9 | 1 | 6.73 | 1405 |
| 4 | 688 | 88 | 4 | 88 | 488 | 9 | 1 | 9.07 | 1405 |
| 4 | 689 | 89 | 4 | 89 | 489 | 9 | 1 | 14.32 | 1405 |
| 4 | 690 | 90 | 4 | 90 | 490 | 9 | 1 | 12.65 | 1405 |
| 4 | 691 | 91 | 4 | 91 | 491 | 10 | 1 | 9.6 | 1405 |
| 4 | 692 | 92 | 4 | 92 | 492 | 10 | 1 | 12.24 | 1405 |
| 4 | 693 | 93 | 4 | 93 | 493 | 10 | 1 | 9.94 | 1405 |
| 4 | 694 | 94 | 4 | 94 | 494 | 10 | 1 | 4.99 | 1405 |
| 4 | 695 | 95 | 4 | 95 | 495 | 10 | 1 | 18.49 | 1405 |
| 4 | 696 | 96 | 4 | 96 | 496 | 10 | 1 | 19 | 1405 |
| 4 | 697 | 97 | 4 | 97 | 497 | 10 | 1 | 18.04 | 1405 |
| 4 | 698 | 98 | 4 | 98 | 498 | 10 | 1 | 4.5 | 1405 |
| 4 | 699 | 99 | 4 | 99 | 499 | 10 | 1 | 0 | 1405 |
| 4 | 700 | 100 | 4 | 100 | 4100 | 10 | 1 | 0 | 1405 |
| 4 | 701 | 1 | 9 | 101 | 41 | 11 | 2 | 18.09 | 1405 |
| 4 | 702 | 2 | 9 | 102 | 42 | 12 | 2 | 32.06 | 1405 |
| 4 | 703 | 3 | 9 | 103 | 43 | 13 | 2 | 24.88 | 1405 |
| 4 | 704 | 4 | 9 | 104 | 44 | 14 | 2 | 13.6 | 1405 |
| 4 | 705 | 5 | 9 | 105 | 45 | 15 | 2 | 15.78 | 1405 |
| 4 | 706 | 6 | 9 | 106 | 46 | 16 | 2 | 3.54 | 1405 |
| 4 | 707 | 7 | 9 | 107 | 47 | 17 | 2 | 22.15 | 1405 |
| 4 | 708 | 8 | 9 | 108 | 48 | 18 | 2 | 7.43 | 1405 |
| 4 | 709 | 9 | 9 | 109 | 49 | 19 | 2 | 14.93 | 1405 |
| 4 | 710 | 10 | 9 | 110 | 410 | 20 | 2 | 23.92 | 1405 |
| 4 | 711 | 11 | 9 | 111 | 411 | 11 | 2 | 27.26 | 1405 |
| 4 | 712 | 12 | 9 | 112 | 412 | 12 | 2 | 14.84 | 1405 |
| 4 | 713 | 13 | 9 | 113 | 413 | 13 | 2 | 16.02 | 1405 |
| 4 | 714 | 14 | 9 | 114 | 414 | 14 | 2 | 14.21 | 1405 |
| 4 | 715 | 15 | 9 | 115 | 415 | 15 | 2 | 8.02 | 1405 |
| 4 | 716 | 16 | 9 | 116 | 416 | 16 | 2 | 17.62 | 1405 |
| 4 | 717 | 17 | 9 | 117 | 417 | 17 | 2 | 13.43 | 1405 |
| 4 | 718 | 18 | 9 | 118 | 418 | 18 | 2 | 17.04 | 1405 |
| 4 | 719 | 19 | 9 | 119 | 419 | 19 | 2 | 18.8 | 1405 |
| 4 | 720 | 20 | 9 | 120 | 420 | 20 | 2 | 15.27 | 1405 |
| 4 | 721 | 21 | 9 | 121 | 421 | 11 | 2 | 32.95 | 1405 |
| 4 | 722 | 22 | 9 | 122 | 422 | 12 | 2 | 11.1 | 1405 |
| 4 | 723 | 23 | 9 | 123 | 423 | 13 | 2 | 9.21 | 1405 |
| 4 | 724 | 24 | 9 | 124 | 424 | 14 | 2 | 5.64 | 1405 |
| 4 | 725 | 25 | 9 | 125 | 425 | 15 | 2 | 15.04 | 1405 |
| 4 | 726 | 26 | 9 | 126 | 426 | 16 | 2 | 25.5 | 1405 |
| 4 | 727 | 27 | 9 | 127 | 427 | 17 | 2 | 26.23 | 1405 |
| 4 | 728 | 28 | 9 | 128 | 428 | 18 | 2 | 8.08 | 1405 |
| 4 | 729 | 29 | 9 | 129 | 429 | 19 | 2 | 25.44 | 1405 |
| 4 | 730 | 30 | 9 | 130 | 430 | 20 | 2 | 9.34 | 1405 |
| 4 | 731 | 31 | 9 | 131 | 431 | 11 | 2 | 8.04 | 1405 |
| 4 | 732 | 32 | 9 | 132 | 432 | 12 | 2 | 11.93 | 1405 |
| 4 | 733 | 33 | 9 | 133 | 433 | 13 | 2 | 27.27 | 1405 |
| 4 | 734 | 34 | 9 | 134 | 434 | 14 | 2 | 6.88 | 1405 |
| 4 | 735 | 35 | 9 | 135 | 435 | 15 | 2 | 5.11 | 1405 |
| 4 | 736 | 36 | 9 | 136 | 436 | 16 | 2 | 17.94 | 1405 |
| 4 | 737 | 37 | 9 | 137 | 437 | 17 | 2 | 17.13 | 1405 |
| 4 | 738 | 38 | 9 | 138 | 438 | 18 | 2 | 14.87 | 1405 |
| 4 | 739 | 39 | 9 | 139 | 439 | 19 | 2 | 19.23 | 1405 |
| 4 | 740 | 40 | 9 | 140 | 440 | 20 | 2 | 27.52 | 1405 |
| 4 | 741 | 41 | 9 | 141 | 441 | 11 | 2 | 15.59 | 1405 |
| 4 | 742 | 42 | 9 | 142 | 442 | 12 | 2 | 14.03 | 1405 |
| 4 | 743 | 43 | 9 | 143 | 443 | 13 | 2 | 17.89 | 1405 |
| 4 | 744 | 44 | 9 | 144 | 444 | 14 | 2 | 32.02 | 1405 |
| 4 | 745 | 45 | 9 | 145 | 445 | 15 | 2 | 7.85 | 1405 |
| 4 | 746 | 46 | 9 | 146 | 446 | 16 | 2 | 10.93 | 1405 |
| 4 | 747 | 47 | 9 | 147 | 447 | 17 | 2 | 25.55 | 1405 |
| 4 | 748 | 48 | 9 | 148 | 448 | 18 | 2 | 7.01 | 1405 |
| 4 | 749 | 49 | 9 | 149 | 449 | 19 | 2 | 2.18 | 1405 |
| 4 | 750 | 50 | 9 | 150 | 450 | 20 | 2 | 7.72 | 1405 |
| 4 | 751 | 51 | 9 | 151 | 451 | 11 | 2 | 13.19 | 1405 |
| 4 | 752 | 52 | 9 | 152 | 452 | 12 | 2 | 13.73 | 1405 |
| 4 | 753 | 53 | 9 | 153 | 453 | 13 | 2 | 22.32 | 1405 |
| 4 | 754 | 54 | 9 | 154 | 454 | 14 | 2 | 14.45 | 1405 |
| 4 | 755 | 55 | 9 | 155 | 455 | 15 | 2 | 21.96 | 1405 |
| 4 | 756 | 56 | 9 | 156 | 456 | 16 | 2 | 11.27 | 1405 |
| 4 | 757 | 57 | 9 | 157 | 457 | 17 | 2 | 9.8 | 1405 |
| 4 | 758 | 58 | 9 | 158 | 458 | 18 | 2 | 20.13 | 1405 |
| 4 | 759 | 59 | 9 | 159 | 459 | 19 | 2 | 15.52 | 1405 |
| 4 | 760 | 60 | 9 | 160 | 460 | 20 | 2 | 7.92 | 1405 |
| 4 | 761 | 61 | 9 | 161 | 461 | 11 | 2 | 19.75 | 1405 |
| 4 | 762 | 62 | 9 | 162 | 462 | 12 | 2 | 10.04 | 1405 |
| 4 | 763 | 63 | 9 | 163 | 463 | 13 | 2 | 20.38 | 1405 |
| 4 | 764 | 64 | 9 | 164 | 464 | 14 | 2 | 18.81 | 1405 |
| 4 | 765 | 65 | 9 | 165 | 465 | 15 | 2 | 8.99 | 1405 |
| 4 | 766 | 66 | 9 | 166 | 466 | 16 | 2 | 10.96 | 1405 |
| 4 | 767 | 67 | 9 | 167 | 467 | 17 | 2 | 16.73 | 1405 |
| 4 | 768 | 68 | 9 | 168 | 468 | 18 | 2 | 14.33 | 1405 |
| 4 | 769 | 69 | 9 | 169 | 469 | 19 | 2 | 18.08 | 1405 |
| 4 | 770 | 70 | 9 | 170 | 470 | 20 | 2 | 29.73 | 1405 |
| 4 | 771 | 71 | 9 | 171 | 471 | 11 | 2 | 9.63 | 1405 |
| 4 | 772 | 72 | 9 | 172 | 472 | 12 | 2 | 25.15 | 1405 |
| 4 | 773 | 73 | 9 | 173 | 473 | 13 | 2 | 27.34 | 1405 |
| 4 | 774 | 74 | 9 | 174 | 474 | 14 | 2 | 4.93 | 1405 |
| 4 | 775 | 75 | 9 | 175 | 475 | 15 | 2 | 20.63 | 1405 |
| 4 | 776 | 76 | 9 | 176 | 476 | 16 | 2 | 7.58 | 1405 |
| 4 | 777 | 77 | 9 | 177 | 477 | 17 | 2 | 6.12 | 1405 |
| 4 | 778 | 78 | 9 | 178 | 478 | 18 | 2 | 14.53 | 1405 |
| 4 | 779 | 79 | 9 | 179 | 479 | 19 | 2 | 15.48 | 1405 |
| 4 | 780 | 80 | 9 | 180 | 480 | 20 | 2 | 18.05 | 1405 |
| 4 | 781 | 81 | 9 | 181 | 481 | 11 | 2 | 20.25 | 1405 |
| 4 | 782 | 82 | 9 | 182 | 482 | 12 | 2 | 24.52 | 1405 |
| 4 | 783 | 83 | 9 | 183 | 483 | 13 | 2 | 15.62 | 1405 |
| 4 | 784 | 84 | 9 | 184 | 484 | 14 | 2 | 17.99 | 1405 |
| 4 | 785 | 85 | 9 | 185 | 485 | 15 | 2 | 10.4 | 1405 |
| 4 | 786 | 86 | 9 | 186 | 486 | 16 | 2 | 4.56 | 1405 |
| 4 | 787 | 87 | 9 | 187 | 487 | 17 | 2 | 14.7 | 1405 |
| 4 | 788 | 88 | 9 | 188 | 488 | 18 | 2 | 18.85 | 1405 |
| 4 | 789 | 89 | 9 | 189 | 489 | 19 | 2 | 22.7 | 1405 |
| 4 | 790 | 90 | 9 | 190 | 490 | 20 | 2 | 13.5 | 1405 |
| 4 | 791 | 91 | 9 | 191 | 491 | 11 | 2 | 1.09 | 1405 |
| 4 | 792 | 92 | 9 | 192 | 492 | 12 | 2 | 2.66 | 1405 |
| 4 | 793 | 93 | 9 | 193 | 493 | 13 | 2 | 22.14 | 1405 |
| 4 | 794 | 94 | 9 | 194 | 494 | 14 | 2 | 7.32 | 1405 |
| 4 | 795 | 95 | 9 | 195 | 495 | 15 | 2 | 23.24 | 1405 |
| 4 | 796 | 96 | 9 | 196 | 496 | 16 | 2 | 17.78 | 1405 |
| 4 | 797 | 97 | 9 | 197 | 497 | 17 | 2 | 13.55 | 1405 |
| 4 | 798 | 98 | 9 | 198 | 498 | 18 | 2 | NA | 1405 |
| 4 | 799 | 99 | 9 | 199 | 499 | 19 | 2 | 2.01 | 1405 |
| 4 | 800 | 100 | 9 | 200 | 4100 | 20 | 2 | 1.55 | 1405 |
| 5 | 801 | 1 | 5 | 1 | 51 | 1 | 1 | 38.925 | 1615 |
| 5 | 802 | 2 | 5 | 2 | 52 | 1 | 1 | 40.554 | 1615 |
| 5 | 803 | 3 | 5 | 3 | 53 | 1 | 1 | 28.257 | 1615 |
| 5 | 804 | 4 | 5 | 4 | 54 | 1 | 1 | 18.951 | 1615 |
| 5 | 805 | 5 | 5 | 5 | 55 | 1 | 1 | 20.547 | 1615 |
| 5 | 806 | 6 | 5 | 6 | 56 | 1 | 1 | 7.172 | 1615 |
| 5 | 807 | 7 | 5 | 7 | 57 | 1 | 1 | 19.76 | 1615 |
| 5 | 808 | 8 | 5 | 8 | 58 | 1 | 1 | 12.426 | 1615 |
| 5 | 809 | 9 | 5 | 9 | 59 | 1 | 1 | 9.423 | 1615 |
| 5 | 810 | 10 | 5 | 10 | 510 | 1 | 1 | 9.02 | 1615 |
| 5 | 811 | 11 | 5 | 11 | 511 | 2 | 1 | 25.963 | 1615 |
| 5 | 812 | 12 | 5 | 12 | 512 | 2 | 1 | 22.465 | 1615 |
| 5 | 813 | 13 | 5 | 13 | 513 | 2 | 1 | 18.118 | 1615 |
| 5 | 814 | 14 | 5 | 14 | 514 | 2 | 1 | 11.571 | 1615 |
| 5 | 815 | 15 | 5 | 15 | 515 | 2 | 1 | 10.459 | 1615 |
| 5 | 816 | 16 | 5 | 16 | 516 | 2 | 1 | 17.934 | 1615 |
| 5 | 817 | 17 | 5 | 17 | 517 | 2 | 1 | 20.828 | 1615 |
| 5 | 818 | 18 | 5 | 18 | 518 | 2 | 1 | 29.043 | 1615 |
| 5 | 819 | 19 | 5 | 19 | 519 | 2 | 1 | 25.043 | 1615 |
| 5 | 820 | 20 | 5 | 20 | 520 | 2 | 1 | 17.173 | 1615 |
| 5 | 821 | 21 | 5 | 21 | 521 | 3 | 1 | 27.79 | 1615 |
| 5 | 822 | 22 | 5 | 22 | 522 | 3 | 1 | 22.657 | 1615 |
| 5 | 823 | 23 | 5 | 23 | 523 | 3 | 1 | 9.136 | 1615 |
| 5 | 824 | 24 | 5 | 24 | 524 | 3 | 1 | 21.213 | 1615 |
| 5 | 825 | 25 | 5 | 25 | 525 | 3 | 1 | 9.843 | 1615 |
| 5 | 826 | 26 | 5 | 26 | 526 | 3 | 1 | 23.081 | 1615 |
| 5 | 827 | 27 | 5 | 27 | 527 | 3 | 1 | 21.039 | 1615 |
| 5 | 828 | 28 | 5 | 28 | 528 | 3 | 1 | NA | 1615 |
| 5 | 829 | 29 | 5 | 29 | 529 | 3 | 1 | 24.621 | 1615 |
| 5 | 830 | 30 | 5 | 30 | 530 | 3 | 1 | 14.879 | 1615 |
| 5 | 831 | 31 | 5 | 31 | 531 | 4 | 1 | 20.246 | 1615 |
| 5 | 832 | 32 | 5 | 32 | 532 | 4 | 1 | 19.575 | 1615 |
| 5 | 833 | 33 | 5 | 33 | 533 | 4 | 1 | 20.412 | 1615 |
| 5 | 834 | 34 | 5 | 34 | 534 | 4 | 1 | 11.817 | 1615 |
| 5 | 835 | 35 | 5 | 35 | 535 | 4 | 1 | 14.702 | 1615 |
| 5 | 836 | 36 | 5 | 36 | 536 | 4 | 1 | 26.515 | 1615 |
| 5 | 837 | 37 | 5 | 37 | 537 | 4 | 1 | 19.118 | 1615 |
| 5 | 838 | 38 | 5 | 38 | 538 | 4 | 1 | 32.532 | 1615 |
| 5 | 839 | 39 | 5 | 39 | 539 | 4 | 1 | 24.152 | 1615 |
| 5 | 840 | 40 | 5 | 40 | 540 | 4 | 1 | 35.318 | 1615 |
| 5 | 841 | 41 | 5 | 41 | 541 | 5 | 1 | 24.214 | 1615 |
| 5 | 842 | 42 | 5 | 42 | 542 | 5 | 1 | 44.877 | 1615 |
| 5 | 843 | 43 | 5 | 43 | 543 | 5 | 1 | 31.027 | 1615 |
| 5 | 844 | 44 | 5 | 44 | 544 | 5 | 1 | 27.095 | 1615 |
| 5 | 845 | 45 | 5 | 45 | 545 | 5 | 1 | 23.746 | 1615 |
| 5 | 846 | 46 | 5 | 46 | 546 | 5 | 1 | 0 | 1615 |
| 5 | 847 | 47 | 5 | 47 | 547 | 5 | 1 | 40.332 | 1615 |
| 5 | 848 | 48 | 5 | 48 | 548 | 5 | 1 | 28.609 | 1615 |
| 5 | 849 | 49 | 5 | 49 | 549 | 5 | 1 | 0 | 1615 |
| 5 | 850 | 50 | 5 | 50 | 550 | 5 | 1 | 42.844 | 1615 |
| 5 | 851 | 51 | 5 | 51 | 551 | 6 | 1 | 32.358 | 1615 |
| 5 | 852 | 52 | 5 | 52 | 552 | 6 | 1 | 14.574 | 1615 |
| 5 | 853 | 53 | 5 | 53 | 553 | 6 | 1 | 24.015 | 1615 |
| 5 | 854 | 54 | 5 | 54 | 554 | 6 | 1 | 30.081 | 1615 |
| 5 | 855 | 55 | 5 | 55 | 555 | 6 | 1 | 24.146 | 1615 |
| 5 | 856 | 56 | 5 | 56 | 556 | 6 | 1 | 14.614 | 1615 |
| 5 | 857 | 57 | 5 | 57 | 557 | 6 | 1 | 24.919 | 1615 |
| 5 | 858 | 58 | 5 | 58 | 558 | 6 | 1 | NA | 1615 |
| 5 | 859 | 59 | 5 | 59 | 559 | 6 | 1 | 12.918 | 1615 |
| 5 | 860 | 60 | 5 | 60 | 560 | 6 | 1 | 10.819 | 1615 |
| 5 | 861 | 61 | 5 | 61 | 561 | 7 | 1 | 19.791 | 1615 |
| 5 | 862 | 62 | 5 | 62 | 562 | 7 | 1 | 13.694 | 1615 |
| 5 | 863 | 63 | 5 | 63 | 563 | 7 | 1 | 14.066 | 1615 |
| 5 | 864 | 64 | 5 | 64 | 564 | 7 | 1 | 23.236 | 1615 |
| 5 | 865 | 65 | 5 | 65 | 565 | 7 | 1 | 12.884 | 1615 |
| 5 | 866 | 66 | 5 | 66 | 566 | 7 | 1 | 24.702 | 1615 |
| 5 | 867 | 67 | 5 | 67 | 567 | 7 | 1 | 19.514 | 1615 |
| 5 | 868 | 68 | 5 | 68 | 568 | 7 | 1 | 35 | 1615 |
| 5 | 869 | 69 | 5 | 69 | 569 | 7 | 1 | 13.853 | 1615 |
| 5 | 870 | 70 | 5 | 70 | 570 | 7 | 1 | 21.288 | 1615 |
| 5 | 871 | 71 | 5 | 71 | 571 | 8 | 1 | 7.763 | 1615 |
| 5 | 872 | 72 | 5 | 72 | 572 | 8 | 1 | 22.271 | 1615 |
| 5 | 873 | 73 | 5 | 73 | 573 | 8 | 1 | 29.477 | 1615 |
| 5 | 874 | 74 | 5 | 74 | 574 | 8 | 1 | 0 | 1615 |
| 5 | 875 | 75 | 5 | 75 | 575 | 8 | 1 | 34.585 | 1615 |
| 5 | 876 | 76 | 5 | 76 | 576 | 8 | 1 | 24.684 | 1615 |
| 5 | 877 | 77 | 5 | 77 | 577 | 8 | 1 | 0 | 1615 |
| 5 | 878 | 78 | 5 | 78 | 578 | 8 | 1 | 21.858 | 1615 |
| 5 | 879 | 79 | 5 | 79 | 579 | 8 | 1 | 14.439 | 1615 |
| 5 | 880 | 80 | 5 | 80 | 580 | 8 | 1 | 15.822 | 1615 |
| 5 | 881 | 81 | 5 | 81 | 581 | 9 | 1 | 12.882 | 1615 |
| 5 | 882 | 82 | 5 | 82 | 582 | 9 | 1 | 25.495 | 1615 |
| 5 | 883 | 83 | 5 | 83 | 583 | 9 | 1 | 15.365 | 1615 |
| 5 | 884 | 84 | 5 | 84 | 584 | 9 | 1 | 17.354 | 1615 |
| 5 | 885 | 85 | 5 | 85 | 585 | 9 | 1 | 20.94 | 1615 |
| 5 | 886 | 86 | 5 | 86 | 586 | 9 | 1 | 11.904 | 1615 |
| 5 | 887 | 87 | 5 | 87 | 587 | 9 | 1 | 15.06 | 1615 |
| 5 | 888 | 88 | 5 | 88 | 588 | 9 | 1 | 10.701 | 1615 |
| 5 | 889 | 89 | 5 | 89 | 589 | 9 | 1 | 12.657 | 1615 |
| 5 | 890 | 90 | 5 | 90 | 590 | 9 | 1 | 20.135 | 1615 |
| 5 | 891 | 91 | 5 | 91 | 591 | 10 | 1 | 10.299 | 1615 |
| 5 | 892 | 92 | 5 | 92 | 592 | 10 | 1 | 16.158 | 1615 |
| 5 | 893 | 93 | 5 | 93 | 593 | 10 | 1 | 20.802 | 1615 |
| 5 | 894 | 94 | 5 | 94 | 594 | 10 | 1 | 19.306 | 1615 |
| 5 | 895 | 95 | 5 | 95 | 595 | 10 | 1 | 30.435 | 1615 |
| 5 | 896 | 96 | 5 | 96 | 596 | 10 | 1 | 19.502 | 1615 |
| 5 | 897 | 97 | 5 | 97 | 597 | 10 | 1 | 28.309 | 1615 |
| 5 | 898 | 98 | 5 | 98 | 598 | 10 | 1 | 16.557 | 1615 |
| 5 | 899 | 99 | 5 | 99 | 599 | 10 | 1 | 0 | 1615 |
| 5 | 900 | 100 | 5 | 100 | 5100 | 10 | 1 | 0 | 1615 |
| 5 | 901 | 1 | 10 | 101 | 51 | 11 | 2 | 30.919 | 1615 |
| 5 | 902 | 2 | 10 | 102 | 52 | 12 | 2 | 36.464 | 1615 |
| 5 | 903 | 3 | 10 | 103 | 53 | 13 | 2 | 20.523 | 1615 |
| 5 | 904 | 4 | 10 | 104 | 54 | 14 | 2 | 22.981 | 1615 |
| 5 | 905 | 5 | 10 | 105 | 55 | 15 | 2 | 11.432 | 1615 |
| 5 | 906 | 6 | 10 | 106 | 56 | 16 | 2 | 8.386 | 1615 |
| 5 | 907 | 7 | 10 | 107 | 57 | 17 | 2 | 15.415 | 1615 |
| 5 | 908 | 8 | 10 | 108 | 58 | 18 | 2 | 23.799 | 1615 |
| 5 | 909 | 9 | 10 | 109 | 59 | 19 | 2 | 13.237 | 1615 |
| 5 | 910 | 10 | 10 | 110 | 510 | 20 | 2 | 12.096 | 1615 |
| 5 | 911 | 11 | 10 | 111 | 511 | 11 | 2 | 17.408 | 1615 |
| 5 | 912 | 12 | 10 | 112 | 512 | 12 | 2 | 19.878 | 1615 |
| 5 | 913 | 13 | 10 | 113 | 513 | 13 | 2 | 25.416 | 1615 |
| 5 | 914 | 14 | 10 | 114 | 514 | 14 | 2 | 17.089 | 1615 |
| 5 | 915 | 15 | 10 | 115 | 515 | 15 | 2 | 6.951 | 1615 |
| 5 | 916 | 16 | 10 | 116 | 516 | 16 | 2 | 19.244 | 1615 |
| 5 | 917 | 17 | 10 | 117 | 517 | 17 | 2 | 9.87 | 1615 |
| 5 | 918 | 18 | 10 | 118 | 518 | 18 | 2 | 20.707 | 1615 |
| 5 | 919 | 19 | 10 | 119 | 519 | 19 | 2 | 14.98 | 1615 |
| 5 | 920 | 20 | 10 | 120 | 520 | 20 | 2 | 14.528 | 1615 |
| 5 | 921 | 21 | 10 | 121 | 521 | 11 | 2 | 21.365 | 1615 |
| 5 | 922 | 22 | 10 | 122 | 522 | 12 | 2 | 14.096 | 1615 |
| 5 | 923 | 23 | 10 | 123 | 523 | 13 | 2 | 3.081 | 1615 |
| 5 | 924 | 24 | 10 | 124 | 524 | 14 | 2 | 9.7 | 1615 |
| 5 | 925 | 25 | 10 | 125 | 525 | 15 | 2 | 15.842 | 1615 |
| 5 | 926 | 26 | 10 | 126 | 526 | 16 | 2 | 18.286 | 1615 |
| 5 | 927 | 27 | 10 | 127 | 527 | 17 | 2 | 12.636 | 1615 |
| 5 | 928 | 28 | 10 | 128 | 528 | 18 | 2 | 11.995 | 1615 |
| 5 | 929 | 29 | 10 | 129 | 529 | 19 | 2 | 27.341 | 1615 |
| 5 | 930 | 30 | 10 | 130 | 530 | 20 | 2 | 15.699 | 1615 |
| 5 | 931 | 31 | 10 | 131 | 531 | 11 | 2 | 18.889 | 1615 |
| 5 | 932 | 32 | 10 | 132 | 532 | 12 | 2 | 13.331 | 1615 |
| 5 | 933 | 33 | 10 | 133 | 533 | 13 | 2 | 12.38 | 1615 |
| 5 | 934 | 34 | 10 | 134 | 534 | 14 | 2 | 10.799 | 1615 |
| 5 | 935 | 35 | 10 | 135 | 535 | 15 | 2 | 5.878 | 1615 |
| 5 | 936 | 36 | 10 | 136 | 536 | 16 | 2 | 20.48 | 1615 |
| 5 | 937 | 37 | 10 | 137 | 537 | 17 | 2 | 29.797 | 1615 |
| 5 | 938 | 38 | 10 | 138 | 538 | 18 | 2 | 19.935 | 1615 |
| 5 | 939 | 39 | 10 | 139 | 539 | 19 | 2 | 27.6 | 1615 |
| 5 | 940 | 40 | 10 | 140 | 540 | 20 | 2 | 32.266 | 1615 |
| 5 | 941 | 41 | 10 | 141 | 541 | 11 | 2 | 32.977 | 1615 |
| 5 | 942 | 42 | 10 | 142 | 542 | 12 | 2 | 40.902 | 1615 |
| 5 | 943 | 43 | 10 | 143 | 543 | 13 | 2 | 26.841 | 1615 |
| 5 | 944 | 44 | 10 | 144 | 544 | 14 | 2 | 38.727 | 1615 |
| 5 | 945 | 45 | 10 | 145 | 545 | 15 | 2 | 21.231 | 1615 |
| 5 | 946 | 46 | 10 | 146 | 546 | 16 | 2 | 13.329 | 1615 |
| 5 | 947 | 47 | 10 | 147 | 547 | 17 | 2 | 30.886 | 1615 |
| 5 | 948 | 48 | 10 | 148 | 548 | 18 | 2 | 17.59 | 1615 |
| 5 | 949 | 49 | 10 | 149 | 549 | 19 | 2 | 7.789 | 1615 |
| 5 | 950 | 50 | 10 | 150 | 550 | 20 | 2 | 32.648 | 1615 |
| 5 | 951 | 51 | 10 | 151 | 551 | 11 | 2 | 24.102 | 1615 |
| 5 | 952 | 52 | 10 | 152 | 552 | 12 | 2 | 20.896 | 1615 |
| 5 | 953 | 53 | 10 | 153 | 553 | 13 | 2 | 21.628 | 1615 |
| 5 | 954 | 54 | 10 | 154 | 554 | 14 | 2 | 38.851 | 1615 |
| 5 | 955 | 55 | 10 | 155 | 555 | 15 | 2 | 24.41 | 1615 |
| 5 | 956 | 56 | 10 | 156 | 556 | 16 | 2 | 14.062 | 1615 |
| 5 | 957 | 57 | 10 | 157 | 557 | 17 | 2 | 11.313 | 1615 |
| 5 | 958 | 58 | 10 | 158 | 558 | 18 | 2 | 23.004 | 1615 |
| 5 | 959 | 59 | 10 | 159 | 559 | 19 | 2 | 18.965 | 1615 |
| 5 | 960 | 60 | 10 | 160 | 560 | 20 | 2 | 11.167 | 1615 |
| 5 | 961 | 61 | 10 | 161 | 561 | 11 | 2 | 28.526 | 1615 |
| 5 | 962 | 62 | 10 | 162 | 562 | 12 | 2 | 12.022 | 1615 |
| 5 | 963 | 63 | 10 | 163 | 563 | 13 | 2 | 9.355 | 1615 |
| 5 | 964 | 64 | 10 | 164 | 564 | 14 | 2 | NA | 1615 |
| 5 | 965 | 65 | 10 | 165 | 565 | 15 | 2 | 10.563 | 1615 |
| 5 | 966 | 66 | 10 | 166 | 566 | 16 | 2 | 28.375 | 1615 |
| 5 | 967 | 67 | 10 | 167 | 567 | 17 | 2 | 27.888 | 1615 |
| 5 | 968 | 68 | 10 | 168 | 568 | 18 | 2 | 26.148 | 1615 |
| 5 | 969 | 69 | 10 | 169 | 569 | 19 | 2 | 11.196 | 1615 |
| 5 | 970 | 70 | 10 | 170 | 570 | 20 | 2 | 10.619 | 1615 |
| 5 | 971 | 71 | 10 | 171 | 571 | 11 | 2 | 13.324 | 1615 |
| 5 | 972 | 72 | 10 | 172 | 572 | 12 | 2 | 27.244 | 1615 |
| 5 | 973 | 73 | 10 | 173 | 573 | 13 | 2 | 23.571 | 1615 |
| 5 | 974 | 74 | 10 | 174 | 574 | 14 | 2 | 5 | 1615 |
| 5 | 975 | 75 | 10 | 175 | 575 | 15 | 2 | 37.738 | 1615 |
| 5 | 976 | 76 | 10 | 176 | 576 | 16 | 2 | 14.308 | 1615 |
| 5 | 977 | 77 | 10 | 177 | 577 | 17 | 2 | 6.342 | 1615 |
| 5 | 978 | 78 | 10 | 178 | 578 | 18 | 2 | 19.273 | 1615 |
| 5 | 979 | 79 | 10 | 179 | 579 | 19 | 2 | 14.723 | 1615 |
| 5 | 980 | 80 | 10 | 180 | 580 | 20 | 2 | 7.685 | 1615 |
| 5 | 981 | 81 | 10 | 181 | 581 | 11 | 2 | 24.23 | 1615 |
| 5 | 982 | 82 | 10 | 182 | 582 | 12 | 2 | 27.593 | 1615 |
| 5 | 983 | 83 | 10 | 183 | 583 | 13 | 2 | 22.499 | 1615 |
| 5 | 984 | 84 | 10 | 184 | 584 | 14 | 2 | 25.649 | 1615 |
| 5 | 985 | 85 | 10 | 185 | 585 | 15 | 2 | 19.635 | 1615 |
| 5 | 986 | 86 | 10 | 186 | 586 | 16 | 2 | 12.859 | 1615 |
| 5 | 987 | 87 | 10 | 187 | 587 | 17 | 2 | 20.438 | 1615 |
| 5 | 988 | 88 | 10 | 188 | 588 | 18 | 2 | 20.905 | 1615 |
| 5 | 989 | 89 | 10 | 189 | 589 | 19 | 2 | 19.002 | 1615 |
| 5 | 990 | 90 | 10 | 190 | 590 | 20 | 2 | 12.823 | 1615 |
| 5 | 991 | 91 | 10 | 191 | 591 | 11 | 2 | 0 | 1615 |
| 5 | 992 | 92 | 10 | 192 | 592 | 12 | 2 | 9.668 | 1615 |
| 5 | 993 | 93 | 10 | 193 | 593 | 13 | 2 | 30.545 | 1615 |
| 5 | 994 | 94 | 10 | 194 | 594 | 14 | 2 | 8.333 | 1615 |
| 5 | 995 | 95 | 10 | 195 | 595 | 15 | 2 | 21.501 | 1615 |
| 5 | 996 | 96 | 10 | 196 | 596 | 16 | 2 | 22.887 | 1615 |
| 5 | 997 | 97 | 10 | 197 | 597 | 17 | 2 | 18.359 | 1615 |
| 5 | 998 | 98 | 10 | 198 | 598 | 18 | 2 | 22.126 | 1615 |
| 5 | 999 | 99 | 10 | 199 | 599 | 19 | 2 | 0 | 1615 |
| 5 | 1000 | 100 | 10 | 200 | 5100 | 20 | 2 | 0 | 1615 |

Harv = Harvests; Id = Identifier; Gen = Genotypes; HarvRep = Concatenation of harvest and repetition effects; Plot = Plots; Int = Interaction; Block = Block; Rep = Repetition; BY = Biomass yield; Day = Harvest day.
