# Supplementary material for: Genetic insights into elephantgrass persistence for bioenergy purpose
Source: PLoS One. 2018 Sep 13;13(9):e0203818. doi: 10.1371/journal.pone.0203818 (PMC6136769; doi:10.1371/journal.pone.0203818)
Supplement: S2 Table — (DOCX) [file pone.0203818.s004.docx]

**S2 Table.** Registrations names of clones of the Active Elephantgrass Germplasm Bank (BAGCE) mantained by Embrapa Dairy Cattle Research Center and their respective code.

| Code ^†^ | BAGCE registration | Code | BAGCE registration | Code | BAGCE registration | Code | BAGCE registration |
| --- | --- | --- | --- | --- | --- | --- | --- |
| 1 | Elefante da Colômbia | 26 | Mineiro | 51 | Guaco | 76 | 12 AD IRI |
| 2 | BAGCE 2 | 27 | Mole de Volta Grande | 52 | Cuba-115 | 77 | 07 AD IRI |
| 3 | Tres Rios | 28 | Porto Rico | 53 | Cuba-116 | 78 | Pasto Panamá |
| 4 | Napier Volta Grande | 29 | Napier | 54 | Cuba-169 | 79 | BAGCE 92 |
| 5 | Mercker Santa Rita | 30 | Mercker Comum | 55 | King Grass | 80 | 09 AD IRI |
| 6 | Pusa Napier Nº 2 | 31 | Terezópolis | 56 | Roxo Botucatu | 81 | 11 AD IRI |
| 7 | Gigante de Pinda | 32 | Taiwan A-26 | 57 | Mineirão IPEACO | 82 | 05 AD IRI |
| 8 | Napier Goiano | 33 | Duro de Volta Grande | 58 | Vruckwona Africano | 83 | 06 AD IRI |
| 9 | Mercker S. E. A. | 34 | Mercker Comum Pinda | 59 | Cameroon | 84 | 01 AD IRI |
| 10 | Taiwan A-148 | 35 | Turrialba | 60 | BAGCE 69 | 85 | 04 AD IRI |
| 11 | Porto Rico 534-B | 36 | Taiwan A-146 | 61 | Guaçu | 86 | 13 AD IRI |
| 12 | Taiwan A-25 | 37 | Cameroon - Piracicaba | 62 | Napierzinho | 87 | 03 AD IRI |
| 13 | Albano | 38 | Taiwan A-121 | 63 | IJ 7125 | 88 | 02 AD IRI |
| 14 | Híbrido Gigante da Colômbia | 39 | Vrukwona | 64 | IJ 7126 | 89 | 08 AD IRI |
| 15 | Pusa Gigante Napier | 40 | T241 Piracicaba | 65 | IJ 7127 | 90 | Pioneiro |
| 16 | Elefante Híbrido 534-A | 41 | BAGCE 50 | 66 | IJ 7136 | 91 | Banhado |
| 17 | Costa Rica | 42 | BAGCE 51 | 67 | IJ 7139 | 92 | Roxo Farroupilha |
| 18 | Cubano de Pinda | 43 | Elefante Cachoeiro Itapemirim | 68 | IJ 7141 | 93 | Roxo de Canguçu |
| 19 | Mercker Pinda | 44 | Sem Pelo | 69 | Goiano | 94 | Roxo do Itassú |
| 20 | Mercker 86 México | 45 | Capim Cana D'África | 70 | CAC 262 | 95 | BRS Capiaçu |
| 21 | Taiwan A-144 | 46 | Kizozi | 71 | Ibitinema | 96 | CNPGL 91-06-3 |
| 22 | Napier S.E.A. | 47 | Gramafante | 72 | Australiano | 97 | CNPGL 96-25-3 |
| 23 | Taiwan A-143 | 48 | Roxo | 73 | BAGCE 82 | 98 | BRS Canará |
| 24 | Pusa Napier Nº 1 | 49 | Mott | 74 | 13 AD | 99 | CNPGL 94-49-6 |
| 25 | Elefante de Pinda | 50 | BAGCE 59 | 75 | 10 AD IRI | 100 | PCM 0701 |

^†^Code of accessions.
