# Supplementary material for: Genetic insights into elephantgrass persistence for bioenergy purpose
Source: PLoS One. 2018 Sep 13;13(9):e0203818. doi: 10.1371/journal.pone.0203818 (PMC6136769; doi:10.1371/journal.pone.0203818)
Supplement: S3 Table — (DOCX) [file pone.0203818.s005.docx]

**S3 Table. ASReml output of all models tested.**

| Model^a^ | Res. Struct | Gen. order | Perm. order | LogL | NEDF | Iterations | LogL convergence | Constraint | p | BIC |
| --- | --- | --- | --- | --- | --- | --- | --- | --- | --- | --- |
| Leg1.1.H | H | 1 | 1 | -2359.32 | 989 | 8 | Converged | P | 3 | 4739.33 |
| Leg2.1.H | H | 2 | 1 | -2323.49 | 989 | 8 | Converged | P | 5 | 4681.46 |
| Leg3.1.H | H | 3 | 1 | -2312.29 | 989 | 8 | Converged | P | 8 | 4679.75 |
| Leg4.1.H | H | 4 | 1 | -2279.99 | 989 | 213 | Converged | P/? | 12 | 4642.74 |
| Leg5.1.H | H | 5 | 1 | -2250.92 | 989 | 201 | Converged | P/? | 17 | 4619.08 |
| Leg1.2.H | H | 1 | 2 | -2342.6 | 989 | 8 | Converged | P | 5 | 4719.68 |
| Leg2.2.H | H | 2 | 2 | -2324.28 | 989 | 9 | Converged | P/B | 7 | 4696.84 |
| Leg3.2.H | H | 3 | 2 | -2312.28 | 989 | 10 | Converged | P/B | 10 | 4693.53 |
| Leg4.2.H | H | 4 | 2 | -2279.12 | 989 | 5000 | Not converged | P/? | 14 | 4654.79 |
| Leg5.2.H | H | 5 | 2 | -2247.21 | 989 | 5000 | Not converged | P/? | 19 | 4625.46 |
| Leg1.3.H | H | 1 | 3 | -2335.11 | 989 | 306 | Converged | P/? | 8 | 4725.39 |
| Leg2.3.H | H | 2 | 3 | -2316.1 | 989 | 312 | Converged | P/? | 10 | 4701.17 |
| Leg3.3.H | H | 3 | 3 | -2310.54 | 989 | 358 | Converged | P/? | 13 | 4710.74 |
| Leg4.3.H | H | 4 | 3 | -2274.72 | 989 | 424 | Converged | P/? | 17 | 4666.68 |
| Leg5.3.H | H | 5 | 3 | -2240.44 | 989 | 283 | Converged | P/? | 22 | 4632.61 |
| Leg1.4.H | H | 1 | 4 | -2316.9 | 989 | 320 | Converged | P/? | 12 | 4716.56 |
| Leg2.4.H | H | 2 | 4 | -2296.12 | 989 | 344 | Converged | P/? | 14 | 4688.79 |
| Leg3.4.H | H | 3 | 4 | -2284.23 | 989 | 343 | Converged | P/? | 17 | 4685.70 |
| Leg4.4.H | H | 4 | 4 | -2266.82 | 989 | 475 | Converged | P/? | 21 | 4678.47 |
| Leg5.4.H | H | 5 | 4 | -2225.56 | 989 | 323 | Converged | P/? | 26 | 4630.43 |
| Leg1.5.H | H | 1 | 5 | -2244.11 | 989 | 53 | Converged | S/P/? | 17 | 4605.46 |
| Leg2.5.H | H | 2 | 5 | -2222 | 989 | 290 | Converged | S/P/? | 19 | 4575.04 |
| Leg3.5.H | H | 3 | 5 | -2207.26 | 989 | 187 | Converged | S/P/? | 22 | 4566.25 |
| Leg4.5.H | H | 4 | 5 | -2191.4 | 989 | 193 | Converged | S/P | 26 | 4562.11 |
| Leg5.5.H | H | 5 | 5 | -2185.62 | 989 | 278 | Converged | P/? | 31 | 4585.04 |
| Leg1.-.H | H | 1 | 0 | -2362.12 | 989 | 8 | Converged | P | 2 | 4738.03 |
| Leg2.-.H | H | 2 | 0 | -2331.94 | 989 | 9 | Converged | P | 4 | 4691.47 |
| Leg3.-.H | H | 3 | 0 | -2322.63 | 989 | 8 | Converged | P | 7 | 4693.54 |
| Leg4.-.H | H | 4 | 0 | -2297.12 | 989 | 219 | Converged | P/? | 11 | 4670.10 |
| Leg5.-.H | H | 5 | 0 | -2275.76 | 989 | 263 | Converged | P/? | 16 | 4661.87 |
| Leg-.1.H | H | 0 | 1 | -2406.62 | 989 | 8 | Converged | P | 2 | 4827.03 |
| Leg-.2.H | H | 0 | 2 | -2386.72 | 989 | 9 | Converged | P | 4 | 4801.03 |
| Leg-.3.H | H | 0 | 3 | -2374.8 | 989 | 283 | Converged | P/? | 7 | 4797.88 |
| Leg-.4.H | H | 0 | 4 | -2343.35 | 989 | 319 | Converged | P/? | 11 | 4762.56 |
| Leg-.5.H | H | 0 | 5 | -2294.58 | 989 | 134 | Converged | P/? | 16 | 4699.51 |
| Leg1.1.D | D | 1 | 1 | -2323.86 | 989 | 14 | Converged | P | 7 | 4696.00 |
| Leg2.1.D | D | 2 | 1 | -2258.64 | 989 | 10 | Converged | P | 9 | 4579.35 |
| Leg3.1.D | D | 3 | 1 | -2235.8 | 989 | 10 | Converged | P | 12 | 4554.36 |
| Leg4.1.D | D | 4 | 1 | -2219.41 | 989 | 10 | Converged | P | 16 | 4549.17 |
| Leg5.1.D | D | 5 | 1 | -2208.08 | 989 | 10 | Converged | P | 21 | 4560.99 |
| Leg1.2.D | D | 1 | 2 | -2286.23 | 989 | 11 | Converged | P | 9 | 4634.53 |
| Leg2.2.D | D | 2 | 2 | -2256.27 | 989 | 13 | Converged | B/P | 11 | 4588.40 |
| Leg3.2.D | D | 3 | 2 | -2227.15 | 989 | 12 | Converged | B/P | 14 | 4550.85 |
| Leg4.2.D | D | 4 | 2 | -2205.11 | 989 | 18 | Converged | B/P | 18 | 4534.36 |
| Leg5.2.D | D | 5 | 2 | -2190.9 | 989 | 12 | Converged | B/P | 23 | 4540.42 |
| Leg1.3.D | D | 1 | 3 | -2256.98 | 989 | 2099 | Converged | B/P | 12 | 4596.72 |
| Leg2.3.D | D | 2 | 3 | -2235.29 | 989 | 189 | Converged | B/P/? | 14 | 4567.13 |
| Leg3.3.D | D | 3 | 3 | -2231.5 | 989 | 104 | Converged | P/? | 17 | 4580.24 |
| Leg4.3.D | D | 4 | 3 | -2203.2 | 989 | 276 | Converged | B/P/? | 21 | 4551.23 |
| Leg5.3.D | D | 5 | 3 | -2188.39 | 989 | 11 | Converged | B/P | 26 | 4556.09 |
| Leg1.4.D | D | 1 | 4 | -2824.91 | 989 | 12 | Convergence failed | ?/P/B | 16 | 5760.17 |
| Leg2.4.D | D | 2 | 4 | 0 | 989 | 42 | Variance structure is not positive definite |  | 18 | 124.14 |
| Leg3.4.D | D | 3 | 4 | -2240.63 | 989 | 5000 | Not converged | B/P/? | 21 | 4626.09 |
| Leg4.4.D | D | 4 | 4 | 0 | 989 | 184 | Variance structure is not positive definite |  | 25 | 172.42 |
| Leg5.4.D | D | 5 | 4 | 0 | 989 | 35 | Variance structure is not positive definite |  | 30 | 206.90 |
| Leg1.5.D | D | 1 | 5 | -2332.36 | 989 | 5000 | Singularity in Average Information Matrix | S/P/B/? | 21 | 4809.55 |
| Leg2.5.D | D | 2 | 5 | -2280.25 | 989 | 5000 | Singularity in Average Information Matrix | S/P/B/? | 23 | 4719.12 |
| Leg3.5.D | D | 3 | 5 | 0 | 989 | 12 | Singularity in Average Information Matrix | S/P/? | 26 | 179.31 |
| Leg4.5.D | D | 4 | 5 | 0 | 989 | 12 | Singularity in Average Information Matrix | S/P/? | 30 | 206.90 |
| Leg5.5.D | D | 5 | 5 | -2188.16 | 989 | 5000 | Not converged | P/? | 35 | 4617.70 |
| Leg1.-.D | D | 1 | 0 | -2325.45 | 989 | 13 | Converged | P | 6 | 4692.28 |
| Leg2.-.D | D | 2 | 0 | -2268.22 | 989 | 11 | Converged | P | 8 | 4591.61 |
| Leg3.-.D | D | 3 | 0 | -2250.74 | 989 | 11 | Converged | P | 11 | 4577.34 |
| Leg4.-.D | D | 4 | 0 | -2235.69 | 989 | 235 | Converged | P/? | 15 | 4574.83 |
| Leg5.-.D | D | 5 | 0 | 0 | 989 | 81 | Variance structure is not positive definite | P/B | 20 | 137.93 |
| Leg-.1.D | D | 0 | 1 | -2372.9 | 989 | 13 | Converged | P | 6 | 4787.18 |
| Leg-.2.D | D | 0 | 2 | -2337.76 | 989 | 11 | Converged | P | 8 | 4730.69 |
| Leg-.3.D | D | 0 | 3 | -2310.83 | 989 | 13 | Converged | P | 11 | 4697.52 |
| Leg-.4.D | D | 0 | 4 | 0 | 989 | 28 | Variance structure is not positive definite | B/P | 15 | 103.45 |
| Leg-.5.D | D | 0 | 5 | -3829.48 | 989 | 6 | Convergence failed | ?/P | 20 | 7796.89 |
| Leg1.1.US | US | 1 | 1 | -2344.95 | 989 | 10 | Converged | P | 17 | 4807.14 |
| Leg2.1.US | US | 2 | 1 | -2311.46 | 989 | 10 | Converged | P | 19 | 4753.96 |
| Leg3.1.US | US | 3 | 1 | -2298.75 | 989 | 11 | Converged | P | 22 | 4749.23 |
| Leg4.1.US | US | 4 | 1 | -2267.65 | 989 | 491 | Converged | P/? | 26 | 4714.61 |
| Leg5.1.US | US | 5 | 1 | -2239.43 | 989 | 196 | Converged | P/? | 31 | 4692.66 |
| Leg1.2.US | US | 1 | 2 | -2331.39 | 989 | 12 | Converged | P | 19 | 4793.82 |
| Leg2.2.US | US | 2 | 2 | -2312.3 | 989 | 11 | Converged | P/B | 21 | 4769.43 |
| Leg3.2.US | US | 3 | 2 | 0 | 989 | 18 | Variance structure is not positive definite |  | 24 | 165.52 |
| Leg4.2.US | US | 4 | 2 | -2267.52 | 989 | 919 | Converged | P/? | 28 | 4728.15 |
| Leg5.2.US | US | 5 | 2 | -2237.36 | 989 | 186 | Converged | P/? | 33 | 4702.31 |
| Leg1.3.US | US | 1 | 3 | 0 | 989 | 878 | Variance structure is not positive definite |  | 22 | 151.73 |
| Leg2.3.US | US | 2 | 3 | -2310.11 | 989 | 5000 | Not converged | P/B/? | 24 | 4785.74 |
| Leg3.3.US | US | 3 | 3 | -2298.38 | 989 | 5000 | Not converged | P/B/? | 27 | 4782.97 |
| Leg4.3.US | US | 4 | 3 | -2265.65 | 989 | 5000 | Not converged | P/B/? | 31 | 4745.10 |
| Leg5.3.US | US | 5 | 3 | 0 | 989 | 60 | Variance structure is not positive definite |  | 36 | 248.28 |
| Leg1.4.US | US | 1 | 4 | -2339.01 | 989 | 5000 | Not converged | P/B/? | 26 | 4857.33 |
| Leg2.4.US | US | 2 | 4 | -2294.08 | 989 | 5000 | Not converged | P/B/? | 28 | 4781.27 |
| Leg3.4.US | US | 3 | 4 | -2285.46 | 989 | 5000 | Not converged | P/B/? | 31 | 4784.72 |
| Leg4.4.US | US | 4 | 4 | 0 | 989 | 11 | Variance structure is not positive definite |  | 35 | 241.38 |
| Leg5.4.US | US | 5 | 4 | 0 | 989 | 803 | Variance structure is not positive definite |  | 40 | 275.87 |
| Leg1.5.US | US | 1 | 5 | -2252.24 | 989 | 795 | Converged | S/P/? | 31 | 4718.28 |
| Leg2.5.US | US | 2 | 5 | -2289.84 | 989 | 5000 | Singularity in Average Information Matrix | S/P/B/? | 33 | 4807.27 |
| Leg3.5.US | US | 3 | 5 | 0 | 989 | 11 | Singularity in Average Information Matrix | S/P/? | 36 | 248.28 |
| Leg4.5.US | US | 4 | 5 | 0 | 989 | 13 | Singularity in Average Information Matrix | S/P/? | 40 | 275.87 |
| Leg5.5.US | US | 5 | 5 | -2174.28 | 989 | 167 | Converged | P/? | 45 | 4658.91 |
| Leg1.-.US | US | 1 | 0 | -2347.5 | 989 | 10 | Converged | P | 16 | 4805.35 |
| Leg2.-.US | US | 2 | 0 | -2317.77 | 989 | 11 | Converged | P | 18 | 4759.68 |
| Leg3.-.US | US | 3 | 0 | -2306.23 | 989 | 11 | Converged | P | 21 | 4757.29 |
| Leg4.-.US | US | 4 | 0 | -2279.92 | 989 | 490 | Converged | P/? | 25 | 4732.26 |
| Leg5.-.US | US | 5 | 0 | -2257.79 | 989 | 242 | Converged | P/? | 30 | 4722.48 |
| Leg-.1.US | US | 0 | 1 | -2391.44 | 989 | 10 | Converged | P | 16 | 4893.23 |
| Leg-.2.US | US | 0 | 2 | -2375.19 | 989 | 10 | Converged | P | 18 | 4874.52 |
| Leg-.3.US | US | 0 | 3 | -2540.27 | 989 | 9 | Converged | ?/B | 21 | 5225.37 |
| Leg-.4.US | US | 0 | 4 | -2543.96 | 989 | 9 | Converged | ?/B | 25 | 5260.34 |
| Leg-.5.US | US | 0 | 5 | -2282.38 | 989 | 53 | Converged | P/? | 30 | 4771.66 |

^a^The models tested are referred to as Leg*m_a_*.*m_p._.x*, where *m_a_* and *m_p_* represent the Legendre's polynomials orders adjusted for genetic and permanent environmental random effects, respectively, and *x* is the homogeneous (H), diagonal (D) or unstructured (US) residual variance structure.

Res. Struct = Residual structure. Homogeneous (H), diagonal (D) or unstructured (US) residual variance structure.

Gen. order = Fitted order for genetic random effect.

Perm. order = Fitted order for permanent environment random effect.

LogL = Logarithm of likelihood function.

NEDF = Residual degrees of freedom

Iterations = Number of iteractions.

LogL convergence = Status of LogL convergence.

Constraint = ASReml warning code for parameters. P = positive definite, B = fixed at a boundary,S = Singular Information matrix,? = liable to change from P to B.

p = Total number of parameters estimated.

BIC = Schwarz Bayesian information criteria.
