# Supplementary material for: Genetic insights into elephantgrass persistence for bioenergy purpose
Source: PLoS One. 2018 Sep 13;13(9):e0203818. doi: 10.1371/journal.pone.0203818 (PMC6136769; doi:10.1371/journal.pone.0203818)
Supplement: S1 Code — (DOCX) [file pone.0203818.s006.docx]

**S1 Code. ASReml code.**

######################################################################

!RENAME !ARGS 1 2 3 4 5// !DOPART $1

Randon Regression BAGCE-BY Homogeneous error

Med

Id

Gen 100 !SORT
 HarvRep

Plot *

Int

Block

Rep 2 !I

BY

Day *

Dados-BAGCE-BS.txt !skip 1 !EMFLAG 5 !AISINGULARITIES !MAXIT 5000

tabulate BY ~ Gen

tabulate BY ~ Plot

!PART 1

!CYCLE 0 1 2 3 4

BY ~ mu Rep leg(Day,4) !r leg(Day,$I).Gen leg(Day,0).Plot !f mv

0 0 2

leg(Day,$I).Gen 2

leg(Day,$I) 0 US !GP

(($I^2+3*$I+2)*0.5)*0

Gen

leg(Day,0).Plot 2

leg(Day,0) 0 US !GP

1

Plot

!PART 2

!CYCLE 0 1 2 3 4

BY ~ mu Rep leg(Day,4) !r leg(Day,$I).Gen leg(Day,1).Plot !f mv

0 0 2

leg(Day,$I).Gen 2

leg(Day,$I) 0 US !GP

(($I^2+3*$I+2)*0.5)*0

Gen

leg(Day,1).Plot 2

leg(Day,1) 0 US !GP

3*0

Plot

!PART 3

!CYCLE 0 1 2 3 4

BY ~ mu Rep leg(Day,4) !r leg(Day,$I).Gen leg(Day,2).Plot !f mv

0 0 2

leg(Day,$I).Gen 2

leg(Day,$I) 0 US !GP

(($I^2+3*$I+2)*0.5)*0

Gen

leg(Day,2).Plot 2

leg(Day,2) 0 US !GP

6*0

Plot

!PART 4

!CYCLE 0 1 2 3 4

BY ~ mu Rep leg(Day,4) !r leg(Day,$I).Gen leg(Day,3).Plot !f mv

0 0 2

leg(Day,$I).Gen 2

leg(Day,$I) 0 US !GP

(($I^2+3*$I+2)*0.5)*0

Gen

leg(Day,3).Plot 2

leg(Day,3) 0 US !GP

10*0

Plot

!PART 5

!CYCLE 0 1 2 3 4

BY ~ mu Rep leg(Day,4) !r leg(Day,$I).Gen leg(Day,4).Plot !f mv

0 0 2

leg(Day,$I).Gen 2

leg(Day,$I) 0 US !GP

(($I^2+3*$I+2)*0.5)*0

Gen

leg(Day,4).Plot 2

leg(Day,4) 0 US !GP

15*0

Plot

######################################################################

!RENAME !ARGS 1 2 3 4 5// !DOPART $1

Randon Regression BAGCE-BY Diagonal error

Med

Id

Gen 100 !SORT
 HarvRep

Plot *

Int

Block

Rep 2 !I

BY

Day *

Dados-BAGCE-BS.txt !skip 1 !EMFLAG 5 !AISINGULARITIES !MAXIT 5000

tabulate BY ~ Gen

tabulate BY ~ Plot

!PART 1

!CYCLE 0 1 2 3 4

BY ~ mu Rep leg(Day,4) !r leg(Day,$I).Gen leg(Day,0).Plot !f mv

5 1 2

200 0 ID

200 0 ID

200 0 ID

200 0 ID

200 0 ID

leg(Day,$I).Gen 2

leg(Day,$I) 0 US !GP

(($I^2+3*$I+2)*0.5)*0

Gen

leg(Day,0).Plot 2

leg(Day,0) 0 US !GP

1

Plot

!PART 2

!CYCLE 0 1 2 3 4

BY ~ mu Rep leg(Day,4) !r leg(Day,$I).Gen leg(Day,1).Plot !f mv

5 1 2

200 0 ID

200 0 ID

200 0 ID

200 0 ID

200 0 ID

leg(Day,$I).Gen 2

leg(Day,$I) 0 US !GP

(($I^2+3*$I+2)*0.5)*0

Gen

leg(Day,1).Plot 2

leg(Day,1) 0 US !GP

3*0

Plot

!PART 3

!CYCLE 0 1 2 3 4

BY ~ mu Rep leg(Day,4) !r leg(Day,$I).Gen leg(Day,2).Plot !f mv

5 1 2

200 0 ID

200 0 ID

200 0 ID

200 0 ID

200 0 ID

leg(Day,$I).Gen 2

leg(Day,$I) 0 US !GP

(($I^2+3*$I+2)*0.5)*0

Gen

leg(Day,2).Plot 2

leg(Day,2) 0 US !GP

6*0

Plot

!PART 4

!CYCLE 0 1 2 3 4

BY ~ mu Rep leg(Day,4) !r leg(Day,$I).Gen leg(Day,3).Plot !f mv

5 1 2

200 0 ID

200 0 ID

200 0 ID

200 0 ID

200 0 ID

leg(Day,$I).Gen 2

leg(Day,$I) 0 US !GP

(($I^2+3*$I+2)*0.5)*0

Gen

leg(Day,3).Plot 2

leg(Day,3) 0 US !GP

10*0

Plot

!PART 5

!CYCLE 0 1 2 3 4

BY ~ mu Rep leg(Day,4) !r leg(Day,$I).Gen leg(Day,4).Plot !f mv

5 1 2

200 0 ID

200 0 ID

200 0 ID

200 0 ID

200 0 ID

leg(Day,$I).Gen 2

leg(Day,$I) 0 US !GP

(($I^2+3*$I+2)*0.5)*0

Gen

leg(Day,4).Plot 2

leg(Day,4) 0 US !GP

15*0

Plot

######################################################################

!RENAME !ARGS 1 2 3 4 5// !DOPART $1

Randon Regression BAGCE-BY Unstructured error

Med

Id

Gen 100 !SORT
 HarvRep

Plot *

Int

Block

Rep 2 !I

BY

Day *

Dados-BAGCE-BS.txt !skip 1 !EMFLAG 5 !AISINGULARITIES !MAXIT 5000

tabulate BY ~ Gen

tabulate BY ~ Plot

!PART 1

!CYCLE 0 1 2 3 4

BY ~ mu Rep leg(Day,4) !r leg(Day,$I).Gen leg(Day,0).Plot !f mv

1 2 2 !ASMV 5 !STEP 0.1

0

leg(Day,4) 0 US !GP

15*0

leg(Day,$I).Gen 2

leg(Day,$I) 0 US !GP

(($I^2+3*$I+2)*0.5)*0

Gen

leg(Day,0).Plot 2

leg(Day,0) 0 US !GP

1

Plot

!PART 2

!CYCLE 0 1 2 3 4

BY ~ mu Rep leg(Day,4) !r leg(Day,$I).Gen leg(Day,1).Plot !f mv

1 2 2 !ASMV 5 !STEP 0.1

0

leg(Day,4) 0 US !GP

15*0

leg(Day,$I).Gen 2

leg(Day,$I) 0 US !GP

(($I^2+3*$I+2)*0.5)*0

Gen

leg(Day,1).Plot 2

leg(Day,1) 0 US !GP

3*0

Plot

!PART 3

!CYCLE 0 1 2 3 4

BY ~ mu Rep leg(Day,4) !r leg(Day,$I).Gen leg(Day,2).Plot !f mv

1 2 2 !ASMV 5 !STEP 0.1

0

leg(Day,4) 0 US !GP

15*0

leg(Day,$I).Gen 2

leg(Day,$I) 0 US !GP

(($I^2+3*$I+2)*0.5)*0

Gen

leg(Day,2).Plot 2

leg(Day,2) 0 US !GP

6*0

Plot

!PART 4

!CYCLE 0 1 2 3 4

BY ~ mu Rep leg(Day,4) !r leg(Day,$I).Gen leg(Day,3).Plot !f mv

1 2 2 !ASMV 5 !STEP 0.1

0

leg(Day,4) 0 US !GP

15*0

leg(Day,$I).Gen 2

leg(Day,$I) 0 US !GP

(($I^2+3*$I+2)*0.5)*0

Gen

leg(Day,3).Plot 2

leg(Day,3) 0 US !GP

10*0

Plot

!PART 5

!CYCLE 0 1 2 3 4

BY ~ mu Rep leg(Day,4) !r leg(Day,$I).Gen leg(Day,4).Plot !f mv

1 2 2 !ASMV 5 !STEP 0.1

0

leg(Day,4) 0 US !GP

15*0

leg(Day,$I).Gen 2

leg(Day,$I) 0 US !GP

(($I^2+3*$I+2)*0.5)*0

Gen

leg(Day,4).Plot 2

leg(Day,4) 0 US !GP

15*0

Plot

#####################################################################
